# Stable Isotope-Assisted Evaluation of Different Extraction Solvents for Untargeted Metabolomics of Plants

**DOI:** 10.3390/ijms17071017

**Published:** 2016-06-28

**Authors:** Maria Doppler, Bernhard Kluger, Christoph Bueschl, Christina Schneider, Rudolf Krska, Sylvie Delcambre, Karsten Hiller, Marc Lemmens, Rainer Schuhmacher

**Affiliations:** 1 Center for Analytical Chemistry, Department of Agrobiotechnology (IFA-Tulln), University of Natural Resources and Life Sciences, Vienna (BOKU), Konrad-Lorenz-Strasse 20, 3430 Tulln, Austria; maria.doppler@boku.ac.at (M.D.); bernhard.kluger@boku.ac.at (B.K.); christoph.bueschl@boku.ac.at (C.B.); christina.schneider1@gmx.net (C.S.); rudolf.krska@boku.ac.at (R.K.); marc.lemmens@boku.ac.at (M.L.); 2 Institute for Biotechnology in Plant Production, Department of Agrobiotechnology (IFA-Tulln), University of Natural Resources and Life Sciences, Vienna (BOKU), Konrad-Lorenz-Strasse 20, 3430 Tulln, Austria; 3 Luxembourg Centre for Systems Biomedicine, University of Luxembourg Campus Belval, Avenue du Swing 6, 4367 Esch-Belval, Luxembourg; sylvie.delcambre@uni.lu (S.D.); karsten.hiller@uni.lu (K.H.)

**Keywords:** ^13^C-labelling, plant metabolomics, sample preparation, *Triticum aestivum*, wheat, untargeted metabolomics

## Abstract

The evaluation of extraction protocols for untargeted metabolomics approaches is still difficult. We have applied a novel stable isotope-assisted workflow for untargeted LC-HRMS-based plant metabolomics , which allows for the first time every detected feature to be considered for method evaluation. The efficiency and complementarity of commonly used extraction solvents, namely 1 + 3 (*v*/*v*) mixtures of water and selected organic solvents (methanol, acetonitrile or methanol/acetonitrile 1 + 1 (*v*/*v*)), with and without the addition of 0.1% (*v*/*v*) formic acid were compared. Four different wheat organs were sampled, extracted and analysed by LC-HRMS. Data evaluation was performed with the in-house-developed MetExtract II software and R. With all tested solvents a total of 871 metabolites were extracted in ear, 785 in stem, 733 in leaf and 517 in root samples, respectively. Between 48% (stem) and 57% (ear) of the metabolites detected in a particular organ were found with all extraction mixtures, and 127 of 996 metabolites were consistently shared between all extraction agent/organ combinations. In aqueous methanol, acidification with formic acid led to pronounced pH dependency regarding the precision of metabolite abundance and the number of detectable metabolites, whereas extracts of acetonitrile-containing mixtures were less affected. Moreover, methanol and acetonitrile have been found to be complementary with respect to extraction efficiency. Interestingly, the beneficial properties of both solvents can be combined by the use of a water-methanol-acetonitrile mixture for global metabolite extraction instead of aqueous methanol or aqueous acetonitrile alone.

## 1. Introduction

Two different concepts in metabolomics can be distinguished, namely targeted and untargeted approaches. Targeted metabolomics approaches aim at measuring a predefined number of substances and, in this respect, sample preparation can be optimised according to the physical and chemical properties of these metabolites. In contrast, untargeted metabolomics approaches try to cover as many metabolites as possible regardless of their identity. Thus, in the latter case, sample preparation steps are generally kept to a minimum in order to achieve maximal metabolite coverage.

On the other hand, sample preparation can have a crucial influence on the outcome of a study (for recent reviews see, e.g., Kim and Verpoorte [1] or Vuckovic [2]). Ideally, the preparation of samples should be unbiased, simple, rapid and reproducible with as few handling steps as possible. Usually, sample treatment starts directly after harvesting, when samples are quenched (by shock-freezing, e.g., in liquid nitrogen or by adding organic solvent) to avoid any changes in the composition of metabolites or their concentration levels. For a comprehensive coverage of the metabolome, the number of extracted metabolites should be as high as possible, which includes a high extraction rate per metabolite. Simultaneous matrix effects such as ion suppression/enhancement in the electrospray ion source of the LC-HRMS instrument have to be kept as low as possible [2].

So far, only a few studies have tried to systematically assess sample extraction procedures which are commonly used in untargeted metabolomics. Efforts were made to determine the influence of different parameters (e.g., solvents, temperature, grinding…) on extraction efficiency, matrix effects and precision of the resulting metabolite abundances. Example studies include human blood plasma [3,4] or serum [5], liver tissue of mice [6] or different microbial cultures [7] and also plants [8,9,10].

In many untargeted plant metabolomics studies, methanol or methanol-water mixtures with or without acidification are used for sample extraction (Table 1). For grapes of *Vitis vinifera* [8] and *Arabidopsis* leaves [10], extraction solvents or methods were compared with the purpose of extracting as many metabolites as possible with the best repeatability. Chloroform was tested in comparison to methanol and water by Theodoridis et al. [8]. The authors reported chloroform as being counterproductive for grapevine metabolomics while t´Kindt and coworkers [10] defined the most suitable solvent for *Arabidopsis* to contain approximately equal amounts of methanol and chloroform and up to 20% water. Moreover, Martin et al. recently evaluated eight different solvents with respect to extract reproducibility, yield, and the number of detected metabolite features. The authors found that the combined use of 70% aqueous ethanol followed by hexane and dichloromethane was best-suited. Interestingly, solvent partitioning using 70% aqueous ethanol followed by hexane and dichloromethane was superoir to parallel single extractions [9]. Despite the fact that acidification of extraction solvents has been reported to promote the inactivation of enzymatic activity and thus to prevent metabolic activity during extraction [20], no clear trend according the use of acidified extraction solvents is evident in the literature. This raises the question of if and how acidified solvents affect the extraction process and metabolite detection. Sample extraction is mostly followed by centrifugation and/or filtration and subsequent LC-HRMS measurement. Some approaches include additional sample preparation steps such as solid phase extraction [8,18] or evaporation of the raw extract and re-dissolution in different solvents [11,15,17,19].

None of these studies were able to distinguish between non-biological and truly biology-derived MS signals, which complicates systematic method optimisation as well as validation of the respective analytical workflows. This situation has changed due to the advent of recently developed stable isotope-assisted methods. Uniformly ^13^C (U-^13^C)-labelled biological samples and native ^12^C samples can, for example, be mixed before LC-HRMS analysis to create labelling-specific isotopolog patterns in the mass spectra, which can be used for global yet specific detection of features derived from biological origin. To this end, several software tools have been developed for LC-HRMS data processing during the last few years such as X13CMS [21], mzMatch-ISO [22], HiTIME [23], IROA—Company (avaliable online: http://iroatech.com, last accessed May 2016), ALLocator [24] and MetExtract [13,25].

Some of these tools have been designed to use globally isotope-enriched biological samples and therefore provide new opportunities such as internal standardisation for improved comparative metabolite quantification, and evaluation of the performance of untargeted metabolomics approaches. This benefit allows, for the first time, direct insight into the metabolic composition of individual biological samples originating, for example, from different plant species, organs or tissues.

In the presented work, we applied a recently developed isotope-assisted metabolomics workflow [13] for the systematic evaluation of different extraction solvents for untargeted metabolomics of plants.

## 2. Results

### 2.1. General Description of Extract Compositions

In this study, we compared the effect of six different extraction mixtures on the LC-HRMS metabolite profiles of green cereal extracts. For this purpose, four organs of flowering wheat plants were milled and extracted with aqueous methanol (M), aqueous acetonitrile (A) and a 1:1 (*v*/*v*) mixture of aqueous methanol and acetonitrile (M/A). In each of the extraction mixtures the amount of water has been standardised to 25% (3 + 1, *v*/*v*). Moreover, each of the three extraction mixtures was prepared with and without 0.1% (*v*/*v*) formic acid, resulting in a total of six different extraction mixtures. Native and U-^13^C-labelled wheat samples were extracted separately, mixed and analysed by LC-HRMS. Measurements were carried out using an LC-LTQ-Orbitrap XL instrument and data analysis was performed with MetExtract as described previously [13]. Analytical features with a characteristic ^12^C-^13^C isotope pattern and the same chromatographic behaviour were grouped to feature groups, each of which represents a distinct metabolite. The characteristics of the most intense features per group were used to describe the respective individual metabolite. Among the metabolites consistently detected in three of three replicates of at least one organ/extraction mixture combination, a fraction of about 5% of the metabolites was found in only one of three and another 5% in two of three replicates of at least any of the other organ/extraction mixture pairings. With respect to extracted ion chromatogram (EIC) peak heights, only 25% of the respective metabolites reached intensity values of 10,000 counts.In contrast, for the consistently found metabolites (three of three replicates) 75% exceed this value of 10,000 which corresponds to a factor of about 2–10 above the limit of detection of the Orbitrap mass analyzer. A graphical illustration of frequency and EIC peak area distributions of metabolites not consistently found in three of three replicates can be found in Figure S1. For the rest of the manuscript, all metabolites had to be detected consistently in three of three replicate extracts of the respective experimental variant to be considered for further data evaluation.

**Figure 1:**
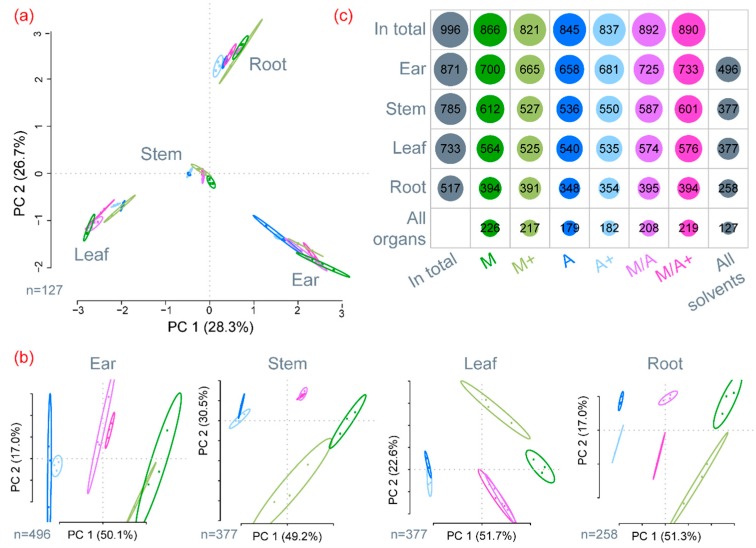
(**a**) Score plot of a principal component analysis employing the peak areas of 127 metabolites detected in all extraction mixtures. The explained variance of the respective principal component is stated in brackets; (**b**) Score plots of principle component analysis for each of the tested wheat organs. In each case PC1 describes about 50% of the variance and extraction mixtures cluster according to solvent composition prior to acidification; (**c**) Overview of the number of metabolites that were detected consistently in the three technical replicates of an extraction mixture and wheat organ. Every column represents the number of metabolites that have been detected by the use of one specific extraction system, and rows represent the respective plant organs.

To identify experimental factors mainly effecting extract composition, the metabolite profiles obtained for all samples and extraction mixtures were evaluated with principle component analysis. The score plot depicted in Figure 1a shows a clear separation of the tested plant organs using only the first two principal components (PC1 and PC2, total covered variance: 55%). Using an additional principal component (PC3) would only further separate the four tested wheat organs and cover a total variance of 71.2% (data not shown). From this, it is clear that the different wheat organs contribute the largest metabolic diversity to the presented study. Thus, for evaluating the effect of extraction mixtures, ear, leaf, stem and root samples were analysed separately. Based on the abundances of the metabolites consistently detected in the organ of interest across the respective extraction mixtures, a clear separation of M, M/A and A along the first principal component was obtained (Figure 1b). Interestingly, in the respective score plots, the position of the samples extracted with M/A was always between the samples extracted with M and A.

In total, 996 different metabolites were detected in the present study. Most metabolites were found in ear samples (871), followed by stem (785) and leaf (733) samples. With a total of 517 substances, roots showed the lowest number of metabolites. Surprisingly, for individual wheat organs the total number of extracted metabolites was roughly the same for every extraction mixture (individual rows in Figure 1c). Although the total number of metabolites was comparable, the metabolite profiles varied significantly between the different extracts, as can also be seen from the fact that only between 48% (377 of 785 in stem) and 57% (496 of 871 in ear) of the detected metabolites in a particular organ were found with all extraction mixtures.

The matching *m*/*z* value and the number of carbon atoms against an in-house wheat database and metabolomics.jp [26] resulted in the annotation of 109 metabolites. Different groups of flavonoids, flavonoid derivatives, amino acids, phenolic acid, hydroxycinnamic acid amides and fatty acid derivatives were annotated. A list with all annotated metabolites is provided in the supplementary material (Table S1).

### 2.2. Precision of Metabolite Abundances

**Figure 2:**
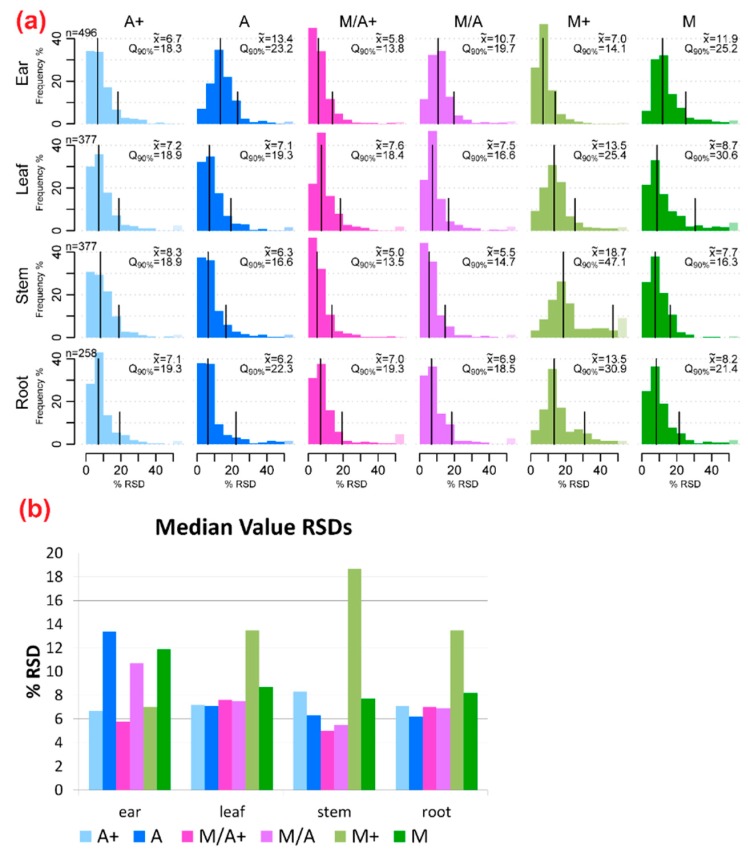
(**a**) Histograms of relative standard deviations (RSDs) of metabolite abundances (area of most intense ^12^C EIC peak per metabolite) for all tested extraction mixture/organ combinations. RSDs were calculated for metabolites extracted with all solvents in the corresponding organ as listed in Figure 1c; (**b**) Diagram of median RSD values for the six tested extraction mixtures, sorted according to wheat organs.

The overall analytical precision was estimated by comparing the distribution of the relative standard deviations (RSDs) between the extraction mixture and wheat organ combinations. For each combination of extraction mixture and plant organ, the RSDs of the metabolite abundances (most intense monoisotopic ^12^C feature per metabolite, no internal standardisation) were calculated and illustrated as histograms (Figure 2).

In general, the precision of EIC peak areas was comparable for the acetonitrile containing extraction agents A, A+, M/A and M/A+ in stem, leaf and root samples (5%–8.3% RSD). For M and M+ the average precision varied strongly between the different organs (7% for M+ in ear to 18.7% for M+ in stem). While M+ resulted in higher RSDs than M for stem, leaf and root samples, for ear samples M+ gave the more precise metabolite abundances.

In the following, differences in the metabolic composition of the generated extracts are presented in more detail. Wheat ears were chosen as an example since the respective extracts contained the highest number of metabolites. The graphs presented for wheat ears in the main text have also been generated for leaf, stem and root samples and can be found in the supplementary material (Figures S2–S10).

### 2.3. Effect of Different Extraction Mixtures on the Metabolic Composition of Wheat Ear Extracts

**Figure 3:**
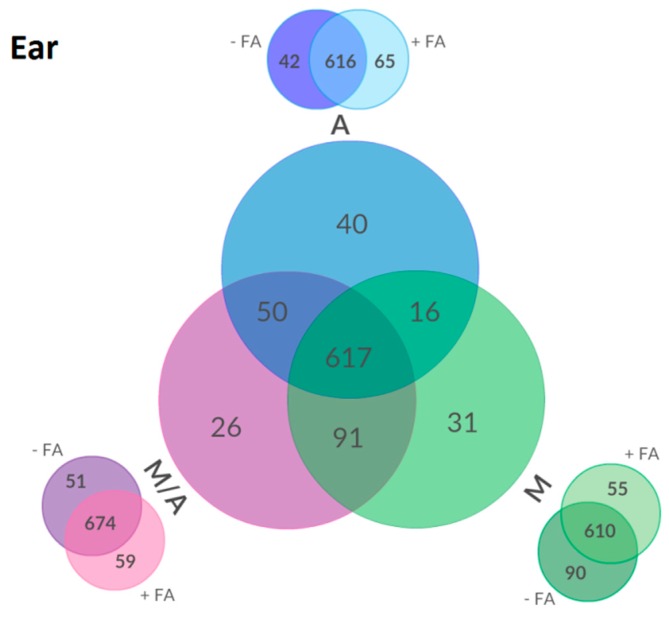
Venn diagram of metabolites detected in ear samples. Metabolite numbers in the large circles refer to the consistent findings (three of three replicates) in at least one type of the respective extraction mixture (non-acidified or acidified form). Small Venn diagrams on the outer side represent the distribution of all metabolites between non-acidified and acidified extraction variants. FA: formic acid.

In total, 871 metabolites were detected in wheat ear samples. As already mentioned above, the total number of metabolites extracted at detectable concentration levels was approximately similar irrespective of the used extraction mixture, but the identity of metabolites as well as their relative abundances was considerably different (Figure 3).

A total of 617 metabolites were present in each of M or M+, A or A+ and M/A or M/A+ generated extracts. The extraction of ear samples with mixture A resulted in the detection of 723 metabolites, while M yielded 755 and M/A 784, respectively. M and M/A shared 91 metabolites which were not detected in samples extracted with A, whereas extracts generated with A and M/A had 50 metabolites in common (not detectable after extraction with M). The low number of 16 metabolites was shared by A- and M-derived extracts only. For ear samples, non-acidified M enabled the detection of 90 more metabolites than M+ whereas, vice versa, only 55 more metabolites could be detected with M+ compared to M. For extracts of M/A vs. M/A+ and A vs. A+, about the same range of metabolites (51 of 59; 42 of 65) could only be detected either in the acidified or non-acidified form of the extraction mixture. For leaf, stem and root samples, similar metabolite distributions were observed as illustrated in the Venn diagrams listed in the supplementary material (Figures S2–S4). The trend towards a lower number of detected metabolites in M+ compared to M could also be observed for stem and leaf samples. For stem samples, for example, a total of 129 metabolites were lost by the acidification of M (see Figure S3).

**Figure 4:**
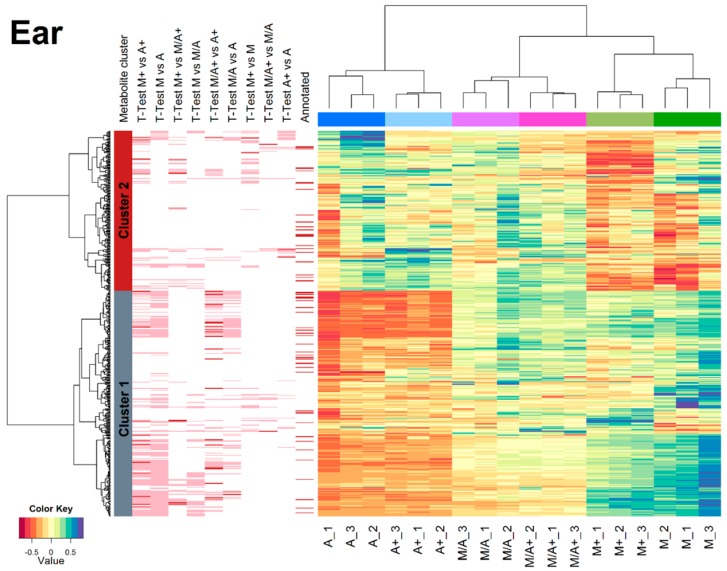
Heatmap, considering all metabolites (*n* = 496) that were consistently extracted from wheat ear samples with all tested extraction mixtures. (EIC peak areas of the native, most intense ion per metabolite were range-scaled and mean-centered. The two dendrograms for the heatmap were calculated using Euclidean distance and ward linkage.) *t*-tests were performed pairwise for the different extraction solvents and significantly different metabolites between two extraction solvents are indicated by a pink bar left of the heatmap (columns “*t*-Test extraction solvent 1 vs. extraction solvent 2”). Metabolites successfully annotated by comparison with databases are indicated in the column “Annotated”. The metabolite dendrogram was cut into two subclusters (red and grey).

The apparent extraction efficiency, which we defined here as the sum of the efficiency with which a particular metabolite is physically extracted and the effect of ion suppression/enhancement, was evaluated by the relative comparison of monoisotopic ^12^C peak areas for those 496 metabolites that were extracted from wheat ears with all of the tested mixtures. The number of commonly found metabolites is lower compared to the 617 denoted in the Venn diagram (Figure 3) since the heatmap only contains metabolites that were detected in each of the six extraction mixtures while the Venn diagram contains metabolites that were found with both as well as either the non-acidified or the acidified form. Based on the 496 shared metabolites, clear differences concerning apparent extraction efficiencies of the tested solvent mixtures were observed when metabolite abundances were compared in a heatmap after two-dimensional hierarchical clustering (Figure 4).

In the heatmap (Figure 4), samples (i.e., columns) are clustered into six groups that correspond to the tested extraction mixtures. Clustering according to differences in acidification was less pronounced but was still consistently observed across all extraction mixtures.

Metabolites illustrated in the rows of the heatmap were clustered into two groups which were characterised by metabolites that were preferentially extracted by M, M+ compared to A, A+ (cluster 1, grey, 290 of 496 metabolites) or A, A+, respectively (cluster 2, red, 206 of 496 metabolites). A minimum mean-fold-change of two and a *p*-value of less than 0.05 were required for a metabolite to be considered as significantly different between two tested extraction mixtures. Cluster 1 (higher abundance after extraction with M/M+) consists of more metabolites that were detected with significantly differing apparent extraction efficiency than cluster 2 which contained metabolites with higher apparent extraction efficiency with A/A+ as the extraction agent.

**Figure 5:**
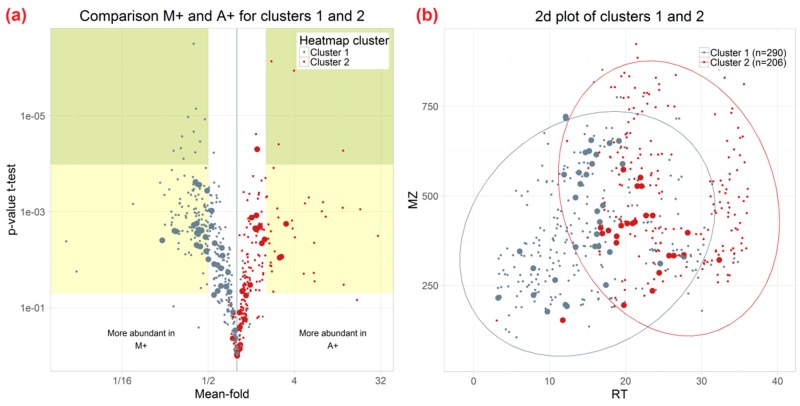
(**a**) Volcano plot of wheat ear metabolites consistently detected with all tested extraction mixtures (cluster 1 grey, cluster 2 red). *p*-values were calculated for the comparison of M+ and A+. Big dots represent annotated metabolites; (**b**) Retention time (RT) vs. *m*/*z* value plot for the same set of metabolites. Grey dots denote metabolites which were extracted with higher apparent efficiency by M+ while red dots refer to metabolites with higher abundance in A+-derived extracts.

The complementary effects of methanol (M/M+) and acetonitrile (A/A+) mixtures on metabolite extraction are also illustrated in the form of a volcano plot (Figure 5).

The two-dimensional (2D) metabolite plot of retention times versus *m*/*z* values further shows differences in the chromatographic behaviour of the metabolites representing the two metabolite clusters (Figure 5b). On average, metabolites with higher apparent extraction efficiency for M+ compared to A+ showed earlier retention times. In contrast, metabolites of cluster 2 showed increased chromatographic retention compared to those of cluster 1 and also tended to have higher *m*/*z* values. In Table 2 those metabolites which significantly differed between M+ and A+ and were annotated, are listed.

For the other tested wheat organs, heatmaps including the *t*-test evaluation of extraction efficiency as well as volcano plots and 2D metabolite plots are provided in the supplementary material (Figures S5–S10). They contain similar clusters with one group holding metabolites that were extracted with higher apparent efficiency by M, M+ compared to A or A+, while another cluster represented metabolites which mostly exhibited higher mass, higher retention times and higher apparent efficiency after extraction with A or A+. In stem, leaf and root samples, pairwise comparison of extraction solvents was performed for M vs. A since in all of these plant organs, the use of non-acidified aqueous methanol resulted in the higher precision of metabolite abundances and a higher number of detected metabolites compared to acidified methanol M+.

## 3. Discussion

### 3.1. General Discussion of Extract Composition and Precision of Metabolite Abundances

This study was aimed at the comparative evaluation of a set of defined solvent mixtures for the extraction of metabolites present in root, stem, leaf and ear samples of flowering wheat plants. We focused on elucidating the number of extractable metabolites, the diversity of metabolite profiles and the precision of technical repeatability, since these parameters are of particular interest to untargeted metabolomics. The applied ^13^C-assisted workflow allowed the efficient filtering of all non-plant-derived LC-HRMS peaks as well as the automated grouping of all features (adduct ions and in source fragments) originating from the same metabolite. By this we were able to consider all truly biology-derived analytical features for the characterization of the plant extracts and the comparison of the different solvent mixtures. In contrast to other studies, which are mainly restricted to the comparison of pre-known substances and/or significantly differing features, e.g., [15,16], it was possible to accurately estimate the total number of detectable metabolites. Since very little noise is contained in the data matrix, our approach also allows the direct comparison of the abundance of all metabolites commonly detected across the investigated extraction mixtures. In this context it is worth mentioning that the type and number of metabolites produced by a particular plant species/organ can considerably differ between genotypes and growth conditions. For this reason, a single wheat genotype (´CM-82036´) has been used. Although the native and highly ^13^C-enriched plants were not grown in parallel under identical conditions, the high number (i.e., overlap) of 996 metabolites found in the investigated extracts demonstrates that both the isotope-assisted approach and samples are well suited for the intended purpose.

Initial data inspection clearly showed that the largest differences with respect to the metabolic composition of sample extracts existed between the four tested wheat organs. This holds true with respect to the total number of detected metabolites, (Figure 1c), as well as the relative abundance of the 127 metabolites shared between all extraction mixture/organ combinations (Figure 1a). Flowering wheat ears were the richest in metabolites, which also reflects the plant’s diverse tissues such as rachis, pedicels, palea, lemma, anthers and stigma.

When comparing the tested extraction variants for each organ separately, we were surprised that for each tissue, the total number of detectable metabolites differed only slightly between extraction mixtures. However, a closer look at the data showed that for a particular organ, only about half of the metabolites were shared consistently across the tested solvent mixtures. Moreover, complementarity of extraction mixtures was also found when the abundances of the common metabolites were used to compare the metabolite profiles by principal component analysis (Figure 1b). In good agreement with the experimental setup, the mixtures M/A and M/A+ were always located between the corresponding aqueous methanol (M and M+) and acetonitrile (A and A+) extracts along the first principal component (PC1).

For untargeted metabolomics workflows, not only the number or diversity of metabolites but also the precision of the measured EIC peak areas is of major interest, since together with biological variability, this parameter may significantly limit the overall precision of any metabolomics approach. With the exception of the acidified aqueous methanol extracts (M+), the median RSD values of the EIC peak areas (most intense monoisotopic native feature per metabolite) ranged from around 6% to 13%, while most of the 90th percentiles were below 25%. Based on these technical precision estimates, the tested solvent mixtures can be considered well suited for global LC-HRMS approaches as the observed RSD values can be expected to be significantly lower than the typical biological variation of metabolite levels [27]. Moreover, the observed technical variations were in good agreement with those reported for other untargeted plant metabolomics workflows. Technical repeatability for untargeted plant metabolomics studies was, for example, on average <20% RSD for grapes [8]; t`Kindt [10] reported 60% of quality control (QC) features to be detected with RSD <20%.

### 3.2. Effect of Acidification of Extraction Mixtures with 0.1% Formic Acid

While the total number of extractable metabolites did not differ much between the tested extraction agents (Figure 3 and Figures S2–S4), there is a slight but general tendency towards a higher metabolite coverage for the three-component mixtures M/A and M/A+ compared to M, M+ or A, A+, respectively. It is worth mentioning that roughly 10% of the detected metabolites were exclusively present in extracts derived from either the non-acidified (A, M/A) or acidified extraction solvents (A+, M/A+). Regarding precision, the median RSDs of root, stem and leaf samples were not influenced by changing the pH in any of the acetonitrile-containing extraction mixtures. However, ear tissues behaved differently as the pH clearly affected the precision of metabolite abundances. Compared to acidified agents, all three non-acidified mixtures showed an increase of median RSDs by a factor of two.

While it is known that acidification inactivates enzyme activity, low pH values in the extraction solvent can be beneficial (e.g., stabilisation of anthocyanins [28]) or disadvantageous such as a reduced number of metabolites or formation of artifacts [20,29]. Moreover, acidification of extraction agents has been reported to affect both extraction efficiency and the stability of phenolics (which can be assumed to constitute a substantial fraction of secondary plant metabolites) in various ways [28]. As illustrated in Table 1, many untargeted metabolomics workflows make use of (acidified) aqueous methanol for metabolite extraction. Therefore, it was also of interest to test how the acidification of methanol affects metabolite coverage and technical precision. Our data indicate that methanol-water extracts behave differently from those generated with acetonitrile-containing solvent mixtures. Acidification of M to M+ resulted in a decrease of the total number of metabolites (*n* = 35 in ear, 85 in stem and 39 in leaf extracts) compared to the non-acidified analogue M. Root samples were an exception to this, as the addition of formic acid to methanol did not affect the number of detectable metabolites. When looking at the relative abundances of commonly extracted metabolites, the heatmap illustrations of individual organs demonstrate that extracts prepared with aqueous methanol (M, M+) were affected most severely by acidification with formic acid. Especially for stem, leaf and root material, the use of acidified aqueous methanol (M+) resulted in lower apparent recoveries compared to M (Figure 4, Figures S5, S7 and S9).

In addition to reduced metabolite coverage and apparent extraction efficiencies, the use of the acidified methanol-water mixture also negatively affected the precision of EIC peak areas of root, leaf and stem metabolites. Compared to non-acidified extracts obtained with M, A or M/A (median RSDs always below 9%), median RSDs of 14%–19% were obtained. Interestingly and contrary to this, the precision of metabolite abundances was not negatively affected when acidified mixtures M+, A+ or M/A+ were used for the extraction of wheat ears (RSDs of 6%–7%).

In order to test whether the measured organ-specific metabolites caused the characteristic pH dependency of precision, RSD histograms were also prepared for those 127 metabolites that were shared between all extraction mixture/organ combinations. Interestingly, for these 127 substances precision patterns showed the same pH dependency across the individual wheat organs (data not shown), suggesting that differences in the composition, permeability or accessibility of the (polymer) sample matrix, rather than the chemical structures of the detected metabolites per se, resulted in characteristic RSD distributions. This complex pH dependency might also result from the varying pH and/or buffer capacity of the extracts generated from the different organs, which deserves being tested in a follow-up study. Moreover, heatmaps generated from the abundances of the same 127 substances also showed organ-specific clusters, which were consistent with those illustrated in Figure 4 and Figures S5, S7 and S9, respectively. Therefore, the apparent extraction efficiencies also seemed to depend on a combined effect of matrix composition and the properties of the respective extraction mixtures rather than the chemical structures of the measured metabolites.

Our results show that neither metabolite coverage nor precision are per se dependent on the extraction agent and that both the biological matrix as well as the extraction agent can influence the precision of metabolite abundances. Similar to this, Mushtaq et al., who reviewed acidification among other extraction methods for metabolomics, could also not observe a clear trend since extraction with acidified solvents was seen as very efficient in one application while in another application extraction efficiency decreased when formic acid was added [30]. In another study, de Vos et al. [31] tested different organic solvents, e.g., methanol, ethanol and acetone, and reported the acidification of a methanol-water mixture (75/25 (*v*/*v*)) with 0.1% FA as most suitable for the efficient extraction of secondary metabolites from plants, while Bertrand et al. [32] observed reduced chemical diversity and a decrease of the number of peaks after the addition of 1% (*v*/*v*) formic acid to extraction solvents.

From the above it can be concluded that the use of acidified aqueous methanol (M+) as an extraction mixture mainly resulted in a decrease of the number of detected metabolites as well as reduced workflow precision. However, despite this general trend, individual extraction agent/plant tissue pairings should be inspected from case to case since exceptions have also been illustrated in our study.

### 3.3. Influence of Extraction Mixture on Relative Abundance of Shared Metabolites

The comparison of the relative apparent extraction efficiencies for the six extraction mixtures was based on those metabolites that were consistently shared between all extracts. For this purpose, plant organs were treated separately again. In each of the heatmaps, two distinct clusters of metabolites (red and grey bars next to the left dendrograms in Figure 4 and Figures S5, S7 and S9) can be observed, which represent metabolites preferentially extracted by aqueous methanol (M and M+) (cluster 1, grey) and another group of metabolites which were more abundant in extracts obtained from aqueous acetonitrile (A and A+) (cluster 2, red). With the exception of leaf extracts, which mainly consisted of metabolites preferentially extracted by aqueous methanol, the two clusters had approximately the same size.

Matching the accurate *m*/*z*, the number of carbon atoms per metabolite ion and predicted ion species against databases resulted in a list of annotated metabolites, significantly differing between M+- and A+-derived wheat ear extracts. In good agreement with their preference for M or A as well as their chromatographic behaviour, metabolites of cluster 1 (grey, higher in M, M+) mainly belonged to phenolic acids, the large group of flavonoids and various glycoside derivatives thereof, whereas metabolites of cluster 2 (red, higher in A, A+) represented more apolar (oxidized) fatty acids and lipids. Since the abundance of polyphenols as well as fatty acids and their oxidized derivatives is frequently altered by various biotic and abiotic perturbations of plants, these differences in apparent extraction efficiencies can be of relevance for the outcome of untargeted metabolomics studies.

Interestingly, all extracts generated from the three-solvent mixtures M/A and M/A+ clustered separately with relative metabolite abundances ranging in between the corresponding aqueous methanol (M, M+) and acetonitrile (A, A+) extracts (Figure 1b, Figure 4 and Figures S5, S7 and S9). These findings suggest that water-methanol-acetonitrile mixtures may be well suited for global metabolite extraction in untargeted plant metabolomics research. The suitability of M/A+ (and M/A) has therefore been further investigated with the example of the ear samples.

**Figure 6:**
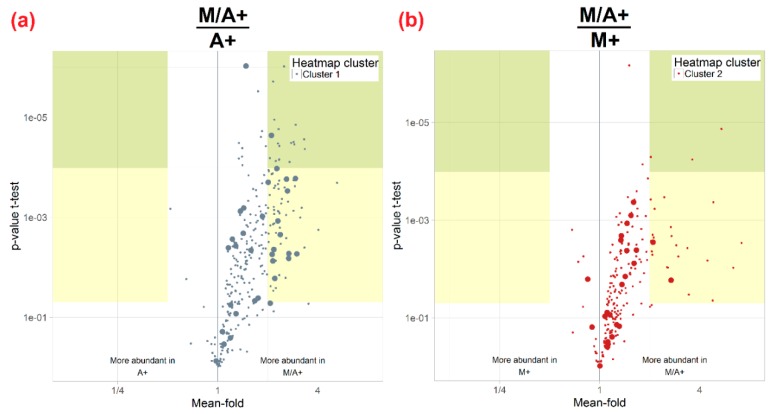
(**a**) Volcano plot, illustrating the abundance of metabolites of cluster 1 (i.e., more efficiently extracted by acidified acetonitrile M+ compared to A+, grey dots) in extracts of the three-solvent mixture M/A+ relative to their abundance in A+-derived extracts; (**b**) Ratios of metabolite abundance and *p*-values of cluster 2 metabolites (primarily extracted by A+, red) measured in M/A+ extracts relative to their abundance after use of M+.

Figure 6a clearly illustrates that those metabolites, which are primarily extracted by acidified aqueous methanol (M+) (grey dots, cluster 1) compared to A+, were also more abundant when a methanol/acetonitrile mixture (M/A+) was used for extraction instead of acidified aqueous methanol alone. At the same time, the abundance of metabolites of cluster 2 (red dots) was not significantly decreased when M/A+ was used for the extraction instead of the more suited A+ (data not shown). Conversely, metabolites originally more abundant after extraction with A+ (red dots, cluster 2) compared to M+ also showed higher peak areas when M/A+ was used for extraction instead of aqueous acetonitrile (A+) alone (Figure 6b). In conclusion, our data demonstrate that the beneficial properties of methanol and acetonitrile can be combined by the use of M/A and M/A+ instead of the two-solvent agents M, M+ or A, A+ alone. Moreover, the general tendency to obtain more metabolites (see discussion above) when plant organs are extracted with M/A or M/A+ further suggests that we use mixtures of water, acetonitrile and methanol instead of aqueous methanol or aqueous acetonitrile alone. The presence of acetonitrile in the extraction mixture did also reduce the marked pH dependency of the metabolite coverage, the apparent extraction efficiency and the precision of metabolite abundances that was observed when aqueous methanol was used as the extraction mixture.

The application of a ^13^C isotope assisted workflow allowed the direct consideration of every detectable metabolite of biological origin which enabled the comprehensive descriptive comparison of different extraction mixtures for four wheat organs. The benefits of this approach can be further utilised and applied not only for different methodical (e.g., sample preparation) questions but also for biological experiments, e.g., for the observation of plant-pathogen interactions or stress response in untargeted plant metabolomics.

## 4. Materials and Methods

### 4.1. Chemicals and Plant Material

Methanol (MeOH, LiChrosolv, LC gradient grade) was purchased from Merck (Darmstadt, Germany); acetonitrile (ACN, HiPerSolv Chromanorm, HPLC gradient grade) from VWR (Vienna Austria). Water was purified successively by reverse osmosis and an ELGA Purelab Ultra-AN-MK2 system (Veolia WateInr, Vienna, Austria); and formic acid (FA, MS grade) was obtained from Sigma-Aldrich (Vienna, Austria). U-^13^C labelled freeze-dried wheat ears, stems, leaves and roots (>97% ^13^C, cultivar “CM-82036”), were obtained from Isolife (Wageningen, The Netherlands).

Authentic reference standards: N-methylanthranilate, 4-triacetate lactone (Merck, Darmstadt Germany), ferulic acid, syringic acid (Fluka, Vienna, Austria), 2,5-dihydroxybenoic acid (Aldrich, Vienna, Austria), methyl-indole-3-carboxylate (Alfa Aesar, Lancashire, UK), indole-3-acetonitrile (MP-Biomedicals, Santa Ana, CA, USA), kaempferol, l-tryptophan (Sigma, Vienna, Austria), alpha linolenic acid (Cayman Europe, Tallin, Estonia), galangin, 3′,4′,5′-*O*-trimethyltricetin, orientin (Extrasynthese, Lyon, France), schaftoside (Phytolab, Vestenbergsgreuth, Germany) and reserpine (Sigma-Aldrich, Vienna, Austria).

HNO_3_, NH_4_NO_3_, Na_2_MoO_4_·2H_2_O, KH_2_PO_4_ and KOH were purchased from Merck (Darmstadt, Germany). Ca(NO_3_)_2_·4H_2_O, Fe-Chelat (C_10_H_12_N_2_NaFeO_3_), MgSO_4_·7H_2_O, MnCl_2_·4H_2_O, ZnSO_4_·7H_2_O and CuSO_4_·5H_2_O were obtained from Sigma-Aldrich (Vienna, Austria) and H_3_BO_3_ from Carl Roth (Karlsruhe, Deutschland). CO_2_ and synthetic air were purchased from Messer (Gumpoldskirchen, Austria).

### 4.2. Cultivation of Wheat

Cultivation of wheat cultivar ´CM-82036´ was performed by use of hydroponics with nutrient solutions based on Hoagland-Stocks [33] in a growing box containing perlite and rock wool. Two plants per pot were grown in a climate chamber with control of temperature and day/night regime. According to the growing stage of the plant day/night light regime was varied between 12 h/12 h and 14 h/10 h. Temperature was set between 14 and 20 °C. Watering regime varied between once a week at the initial growing period and was adapted to two to three times per week according to the uptake of the respective plant. Plants were grown between 60 and 70 days till the flowering stage.

### 4.3. Sampling and Pretreatment

At flowering, plants were cut with scissors and ear, leaf, stem and root tissue was separated and immediately frozen in liquid nitrogen and stored at −80 °C until further processing. Native wheat organs were freeze dried to a remaining water content <5% and stored at −80 °C until further processing. U-^13^C-labelled wheat organs were purchased in freeze-dried form. Sample preparation for LC-HRMS analysis was carried out according to [31] with slight modifications as reported in [34]. Native and U-^13^C-labelled wheat organs were extracted in parallel with the mixtures listed in Table 3. Ear, stem, leaf, and root samples were prepared separately for all presented experiments as described in the following. Native and U-^13^C-labelled organs were milled separately in pre-cooled 50 mL grinding jars (Retsch) for 30 s at 30 Hz employing a ball mill (MM301 Retsch, Haan, Germany). Homogenised wheat powder was stored at −80 °C until further processing.

### 4.4. Extraction with Different Extraction Mixtures

Six different extraction mixtures were prepared from two organic solvents, water and formic acid (Table 3). To this end, either MeOH, ACN or MeOH/ACN 1 + 1 (*v*/*v*) were mixed with water (3 + 1, *v*/*v*) with and without addition of 0.1% formic acid (*v*/*v*) and cooled to 4 °C until further use. For sample extraction three portions of 30 ± 1.2 mg (stdev) of either homogenised native ^12^C wheat roots, stems or leaves were weighed into 2 mL-Eppendorf tubes resulting in a total of three replicates for each extraction solvent and plant organ. For ear samples 20 ± 1.0 mg (stdev) of homogenised material was used. Next, 70 µL (47 µL for ears) of pre-cooled purified water (4 °C) were added to frozen homogenized and freeze dried sample aliquots to achieve a water content which is similar to freshly sampled plant material. In order to minimize the risk of enzyme reactivation, the contact time of pre-cooled sample aliquots with pure water was limited to about 10–15 s by immediate vortexing for a few seconds and direct addition of 1 mL (670 µL for ears) of pre-cooled extraction mixture (M, A, M/A, M+, A+, M/A+). Samples were vortexed for another 10 s and kept in an ultrasonic bath (frequency 47 kHz, power: 105 W) for 15 min. Subsequently samples were centrifuged for 10 min at 14,000 rpm at 4 °C. For each extraction mixture and organ one batch of homogenised U-^13^C-labelled plant material was prepared in parallel.

^13^C sample extracts were pooled by mixing 1 + 1 + 1 + 1 (*v*/*v*/*v*/*v*) ear, leaf, stem and root respectively. For every organ, internal standardisation was achieved by adding the same volume of pooled U-^13^C-labelled extract to the corresponding native extract resulting in (1 + 1, *v*/*v*) mixtures of ^12^C and U-^13^C raw extract. Water or water + 0.1% formic acid (*v*/*v*) was used to reduce the organic solvent(s) to 50% (*v*/*v*). All samples were rigorously vortexed for 10 s and after another centrifugation step (10 min at 14,000 rpm at 4 °C) the supernatants were transferred into 2 mL glass crimp vials for LC-HRMS measurements.

### 4.5. LC-HRMS Analysis

All prepared sample extracts were analysed by LC-HRMS as described earlier [13]. In brief, a UHPLC system (Accela, Thermo Fisher Scientific, San Jose, CA, USA) equipped with a reversed-phase XBridge C_18_, 150 × 2.1 mm i.d., 3.5 µm particle size (Waters, Milford, MA, USA) analytical column as well as water containing 0. % FA (*v*/*v*) (eluent A) and MeOH containing 0.1% FA (*v*/*v*) (eluent B) were used for linear gradient elution starting with 90% A and continuous increase of B up to 100% in 30 min after an initial hold time of 2 min. The UHPLC system was coupled to an LTQ Orbitrap XL (Thermo Fisher Scientific) equipped with an ESI source. All measurements were performed in the positive ionisation mode with a scan range of *m*/*z* 100–1000 and a resolving power setting of 60,000 full width at half maximum (FWHM) at *m*/*z* 400.

For quality control (QC), a standard solution consisting of 15 authentic reference standards (*N*-methylanthranilate, ferulic acid, 2.5-dihydroxybenoic acid, syringic acid, methyl-indole-3-carboxylate, indole-3-acetonitrile, kaempferol, 4-triacetate lactone, l-tryptophan, alpha linolenic acid, galangin, 3′,4′,5′-*O*-trimethyltricetin, orientin, schaftoside and reserpine) each in a concentration of 1 mg/L, dissolved in MeOH + H_2_O 1 + 1 (*v*/*v*) + 0.1% formic acid was prepared and measured every eighth injection throughout each LC-HRMS sequence. Retention time stability, peak area precision as well as mass accuracy were determined for all QC samples to verify proper measurement performance throughout the whole sequence with the help of QCScreen [35] (data not shown).

### 4.6. Data Processing

#### 4.6.1. LC-HRMS Data Processing

LC-HRMS data files were centroided and converted to the mzXML format using the ProteoWizard package [36,37]; version 3.0.5533. The data files were then processed with an updated version of the MetExtract software [13], MetExtract II (manuscript in preparation). Briefly summarized, each metabolite was verified using (I) the unique isotope patterns of the native and labelled isotopologs (intensity threshold: 5000 counts, Δ*m*/*z* error: 3 ppm, max. isotopolog ratio deviation: 25%) and (II) their highly similar chromatographic peaks (XIC-window: 5 ppm, min. number of scans: 3, min. peak correlation (Pearson): 0.75). Moreover, each detected feature pair denoting a certain ion species of a metabolite (e.g., different adducts, in-source fragments) was annotated with its total number of carbon atoms as derived from the *m*/*z* difference between the monoisotopic native and its uniformly ^13^C-labelled ion. Subsequently, different ions of the same metabolite were convoluted into feature groups again using their highly similar chromatographic peak profiles (min. peak correlation (Pearson): 0.9). All detected metabolites were bracketed among the 72 samples using their (a) monoisotopic *m*/*z* (max. deviation: 10 ppm); (b) retention time (max. deviation ± 0.5 min) and (c) their annotated number of carbon atoms. Features missed during initial data processing due to low abundances were searched for in a targeted manner and respective chromatographic peaks were directly integrated without searching for native (M + 1) and ^13^C-labelled (M′ − 1) isotopologs. For all further data analysis steps EIC peaks of the most intense monoisotopic ^12^C ion per metabolite (which could also be a fragment) was integrated and used as a measure of metabolite abundance.

The generated data matrix consisted of meta-information (unique ID, *m*/*z*, retention time (RT), number of carbon atoms, charge state, ion species annotation when possible) of each detected metabolite as well as their abundances (EIC peak area of the native and ^13^C-labeled metabolite forms) in the different samples. Detected metabolite ions were annotated with possible sum formulas (used elements: C, H, O, N, P and S, max. ppm error: 5 ppm) and a database search (databases: metabolomics.jp [26] and an in-house wheat-specific databases, max. ppm error: 5ppm; feature with the highest *m*/*z* value was used) was conducted. For statistical analysis only one feature pair per metabolite was used. For this, the feature pair detected with the highest average abundance across all samples was used. 

#### 4.6.2. Statistical Analysis

Statistical analysis of the generated data matrix was performed with R [38]; version 3.1.0 and R-Studio (available online: https://www.rstudio.com, version 0.97.551, last accessed November 2015). Only the peak areas of the most intense monoisotopic, native isotopologs of the detected metabolites were used. For uni- and multi-variate analysis of the dataset only those metabolites detected continuously in all samples of the respective wheat organ/extraction mixture combination were used. Individual metabolite levels were range-scaled and mean-centered according to van den Berg [39]. A maximum significance threshold of 0.05 and a minimum mean-fold-change of 2 between two extraction mixtures was required for a metabolite to be designated as significant (yellow and green areas in the volcano plots). Moreover, the multiple-testing correction method of Šidák was applied to designate a metabolite as highly-significant (green area in the volcano plots) [40]. Hierarchical cluster analysis (HCA) and heatmap analysis was calculated using Euclidean distance and ward-linkage.

## Figures and Tables

**Table 1:** Overview of selected untargeted LC-MS-based plant metabolomics studies.

Plant (Organ)	Purpose of Study	Fresh or Dried/Extraction Solvent ^1^	Clean up ^1^	Instrument	Reference
Rice (kernels)	To explore the molecular background of quality traits in rice by predictive models based on high-coverage metabolomics	Fresh/MeOH:H_2_O (5:95)	Dilution in 0.1% acetic acid solution, filtration, evaporation and dissolving in H_2_O	LC-QTOF-MS, IT-MS	[11]
Barley	To study the Fusarium infection of barley	Fresh/MeOH:H_2_O (50:50) (untargeted) and ACN:H_2_O (84:16) (targeted for mycotoxins DON and D3G)	Filter (0.22 µm)	UHPLC–QTOF-MS	[12]
Wheat, maize (ears at flowering stage, kernels)	Development of a novel stable isotope labelling-assisted workflow for improved untargeted LC–HRMS	Fresh/MeOH:H_2_O (75:25) + 0.1% FA	Dilution to final ratio MeOH:H_2_O 1:1 + 0.1% FA	HPLC-ESI-Orbitrap	[13]
Brassica vegetables (leaves)	Identification of factors influencing glucosinolate thermal degradation rates	Freeze-dried/MeOH:H_2_0 (75:25) + 0.1% FA	Filter (0.2 µm)	HPLC-QTOF	[14]
*Vitis vinifera* (grapes)	Solvent extraction protocol optimisation	Fresh/MeOH:H_2_O:CHCl_3_ (17 different mixtures)	SPE for aqueous fraction	RP- and HILIC-UPLC-TOF	[8]
*Symphonia globulifera* (leaves, roots, latex, bark, seeds, pericarps and flowers)	Comparative LC-MS-based metabolite profiling	Dried/MeOH	After evaporation ethyl acetate and H_2_O, dried, reconstituted in MeOH	UHPLC G2-HDMS	[15]
*Aconitum* (root)	Analysis of constituents in the root	Fresh/MeOH:H_2_O (75/25)	Filter (0.22 µm)	UHPLC–QTOF–HDMS	[16]
*Medicago x varia*; *Knautia arvensis*; *Lotus corniculatus*; *Bellis perennis*, *Leontodon autumnalis* (leaves)	Contrasting effects of biodiversity on the performance of individual plant species	Fresh/MeOH	Drying, redissolved in 50% MeOH	UHPLC-FT-ICR-MS	[17]
*Rhus typhina* L., *Lythrum salicaria* L., *Monarda Fistulosa* L. (aerial tissue; berries, leaves, stem, flowers, buds)	Evaluation of solvent extraction systems	Dried/eight solvent systems (hexane, dichlormethane, ethyl acetate, methanol, isopropanol, water aqueous ethanol (70%), dichloromethane-methanol (50:50); and additional extract partitioning	For some samples extract partitioning with hexane and dichloromethane	UPLC-ESI-SQ-MS	[9]
*Arabidopsis thaliana* (leaves)	Joint GC- and LC-MS platforms and evaluation of repeatability and sample pre-treatment	Fresh/MeOH:H_2_O (80:20) and CHCl_3_:MeOH:H_2_O (20:60:20) with various protocols	Various protocols	LC-QTOF-MS	[10]
*Arabidopsis thaliana* (leaves)	A systematic comparison of high-resolution quadrupole-time-of-flight and single-stage Orbitrap mass spectrometers	Fresh/IPA:FA (99.5:0.5)	Evaporation, resuspended in MeOH:H_2_O, SPE (C18) MeOH:H_2_O 80:20	UHPLC-QTOF, UHPLC-Exactive Orbitrap	[18]
*Arabidopsis thaliana* (rosette leaves)	Prediction of pathways and novel chemical structures	Fresh/MeOH:H_2_O (80:20)	Dried, redissolved in MeOH:H_2_O (50:50), filter (STAGE tip), remove lipids with chloroform	LC-HRMS Orbitrap	[19]

^1^ Abbreviations: MeOH: methanol; H_2_O: water; ACN: acetonitrile; DON: deoxynivalenol, D3G: DON-3-glucoside, FA: formic acid; CHCl_3_: chloroform; IPA: isopropanol.

**Table 2:** Metabolites significantly differing between A+- and M+-derived wheat ear samples and that were annotated by matching accurate *m*/*z* and number of carbon atoms.

ID	Accurate Mass *m*/*z* of Most Abundant Feature	RT (min)	Cluster	Name or Substance Class	Assigned Molecular Formula	Number of db Hits	*p*-Value M+ vs. A+	Fold Change A+/M+
1	215.1394	3.22	1	d-Desthiobiotin	C_10_H_18_N_2_O_3_	1	0.0025	0.2
12	298.0975	7.84	1	5′-Deoxy-5′-(methylthio)adenosine *	C_11_H_15_N_5_O_3_S	1	0.0039	0.2
13	223.1080	7.88	1	5-Methoxy-3-indoleacetic acid or DL-Indole-3-lactic acid	C_11_H_11_NO_3_	2	0.0026	0.2
17	177.0547	9.69	1	Chlorogenic acid *	C_16_H_18_O_9_	1	0.0053	0.4
29	559.1792	11.9	1	Tetrahydroxyprenylflavanone-hexoside, e.g., Phellavin	C_26_H_32_O_12_	4	0.0004	0.5
31	196.0607	12.08	1	2-Carboxy-2,3-dihydro-5,6-dihydroxyindole or Dopaquinone	C_9_H_9_NO_4_	2	0.0025	0.4
32	721.2328	12.11	1	Dihydrophelloside	C_32_H_42_O_17_	1	0.0028	0.4
45	565.1559	13.86	1	Schaftoside *	C_26_H_28_O_14_	85	0.0019	0.4
46	533.1636	14.03	1	Plumerubroside	C_24_H_30_O_12_	1	0.0017	0.4
48	621.2158	14.71	1	Flavonoid-dihexoside-hydroxycinnamicacid ester, e.g., Petunoside	C_37_H_38_O_19_	9	0.0003	0.4
49	559.1790	14.83	1	Tetrahydroxyprenylflavanone-hexoside, e.g., Phellavin	C_26_H_32_O_12_	4	0.0029	0.4
55	655.1873	15.46	1	Dimethoxy-tetrahydroxyflavon-dihexoside, e.g., Limocitrin 3-rutinoside	C_29_H_34_O_17_	13	0.0024	0.4
60	359.1317	15.96	1	Dihydroxyflavan-hexoside, e.g., Koaburanin	C_21_H_24_O_8_	3	0.0002	0.4
61	615.2264	16.14	1	Flavonoid-trihexoside, e.g., Tricin 7-rutinoside-4′-glucoside	C_35_H_44_O_21_	2	0.0066	0.5
75	647.1590	17.88	1	Methoxy-trihydroxyflavanol-dihexoside or dimethoxy,trihydroxyflavon-di-C-hexoside, e.g., 6-C-Arabinopyranosyl-8-C-glucopyranosyltricin	C_28_H_32_O_16_	75	0.0033	0.5
101	333.2042	26.34	2	9-Hydroperoxy-10,12,15-octadecatrienoate or isomer	C_18_H_30_O_4_	1	0.0018	3
103	335.2199	27.65	2	octadecanoic acid derivatives, e.g., (9*Z*,11*E*)-(13*S*)-13-Hydroperoxyoctadeca-9,11-dienoic acid	C_18_H_32_O_4_	4	0.0086	3.4
108	321.2403	32.31	2	*cis*-9,10-Epoxystearic acid	C_18_H_34_O_3_	1	0.0090	3.6

* confirmed with authentic reference standard.

**Table 3:** Composition of extraction mixtures used in this study.

Extraction Mixture	Organic Solvent (vol)	Water (vol)	Formic Acid (vol)
M	Methanol (75%)	25%	-
A	Acetonitrile (75%)	25%	-
M/A	Methanol/acetonitrile (37.5%/37.5%)	25%	-
M+	Methanol (75%)	25%	0.1%
A+	Acetonitrile (75%)	25%	0.1%
M/A+	Methanol/acetonitrile (37.5%/37.5%)	25%	0.1%

## Supplementary Materials

Supplementary materials can be found at http://www.mdpi.com/1422-0067/17/7/1017/s1.

## Abbreviations

A	extraction mixture (acetonitrile 75%, water 25%)
A+	extraction mixture (acetonitrile 75%, water 25%, formic acid: 0.1%)
ACN	acetonitril
CHCl_3_	chloroform
DAD	diode array detector
DB	database
DON	deoxynivalenol
D3G	DON-3-glucoside
EIC	extracted ion chromatogram
ESI	electrospray ionisation
FA	formic acid
GC	gas chromatography
HCA	hierarchical cluster analysis
HDMS	high definition mass spectrometry
HILIC	hydrophilic interaction liquid chromatography
H_2_O	water
ICR	ion cyclotron resonance
IM	ion mobility
IPA	isopropanol
IT	ion trap
LC	liquid chromatography
LC-HRMS	liquid chromatography high resolution mass spectrometry
LTQ	linear trap quadrupole
MeOH	methanol
MS	mass spectrometry
M	extraction mixture (methanol 75%, water 25%)
M+	extraction mixture (methanol 75%, water 25%, formic acid: 0.1%)
M/A	extraction mixture (methanol 37,5%, acetonitrile 37,5%, water 25%)
M/A+	extraction mixture (methanol 37,5%, acetonitrile 37,5%, water 25%, formic acid: 0.1%))
RP	reversed phase
PC	principle component
PCA	principle component analysis
PDA	photodiode array
QC	quality control
RSD	relative standard deviation
SQ	single quadrupole
(U)HPLC	(ultra) high performance liquid chromatography
UFLC	ultra fast liquid chromatography
