# Supplementary material for: Stable Isotope-Assisted Evaluation of Different Extraction Solvents for Untargeted Metabolomics of Plants
Source: Int J Mol Sci. 2016 Jun 28;17(7):1017. doi: 10.3390/ijms17071017 (PMC4964393; doi:10.3390/ijms17071017)
Supplement: Supplementary file 1 [file ijms-17-01017-s001.pdf]

Supplementary Materials: Stable Isotope-Assisted Evaluation of Different Extraction Solvents for Untargeted Metabolomics of Plants

Maria Doppler, Bernhard Kluger, Christoph Bueschl, Christina Schneider, Rudolf Krska, Sylvie Delcambre, Karsten Hiller, Marc Lemmens and Rainer Schuhmacher

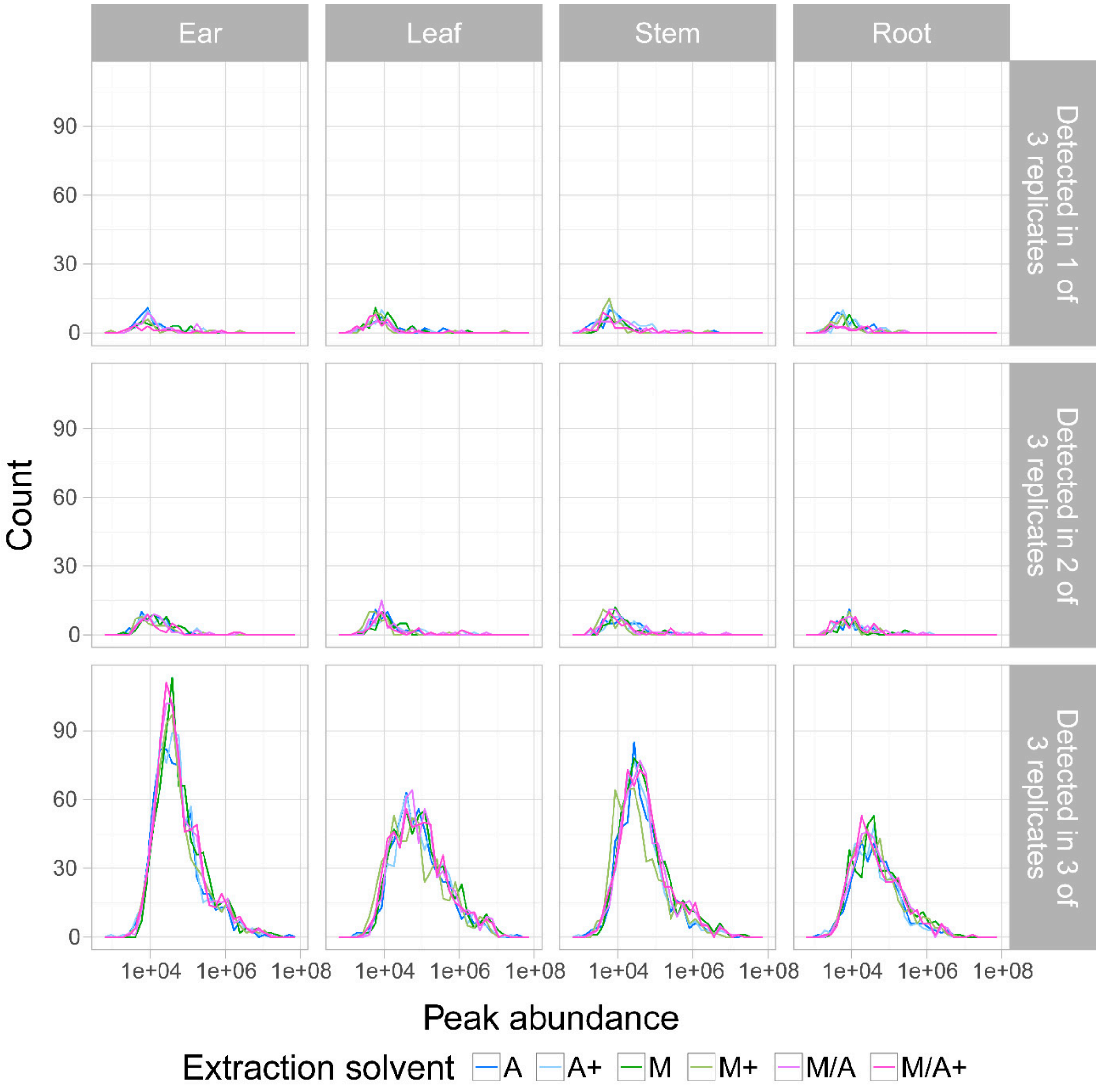

Figure S1. Intensity distributions of metabolites found in one, two or three of three replicates.

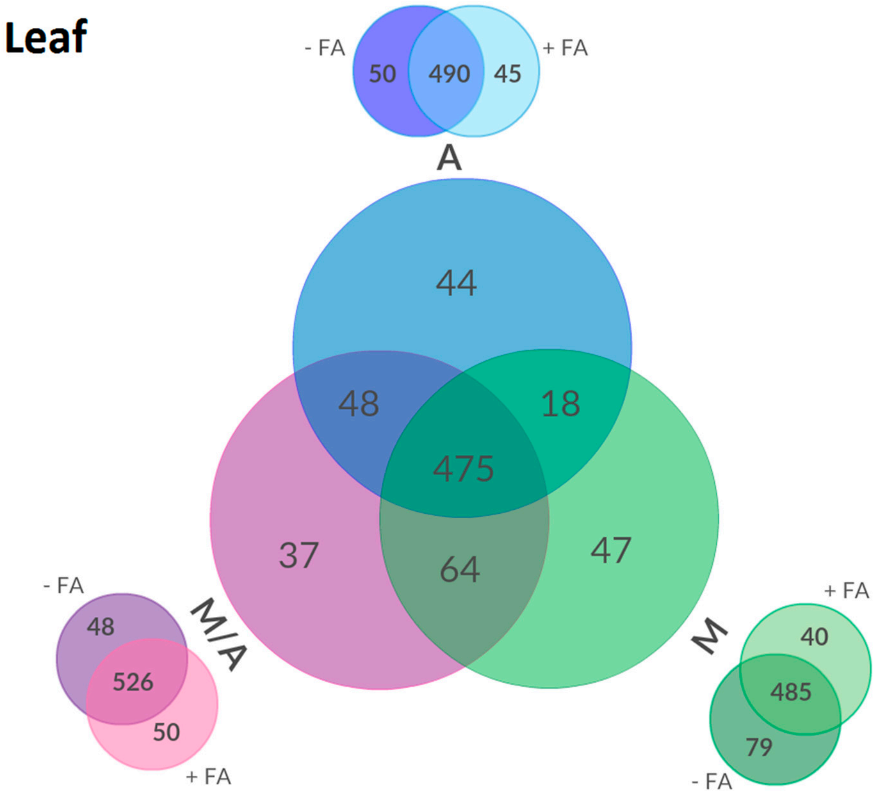

Figure S2. Venn diagram of metabolites detected in wheat leaf samples.

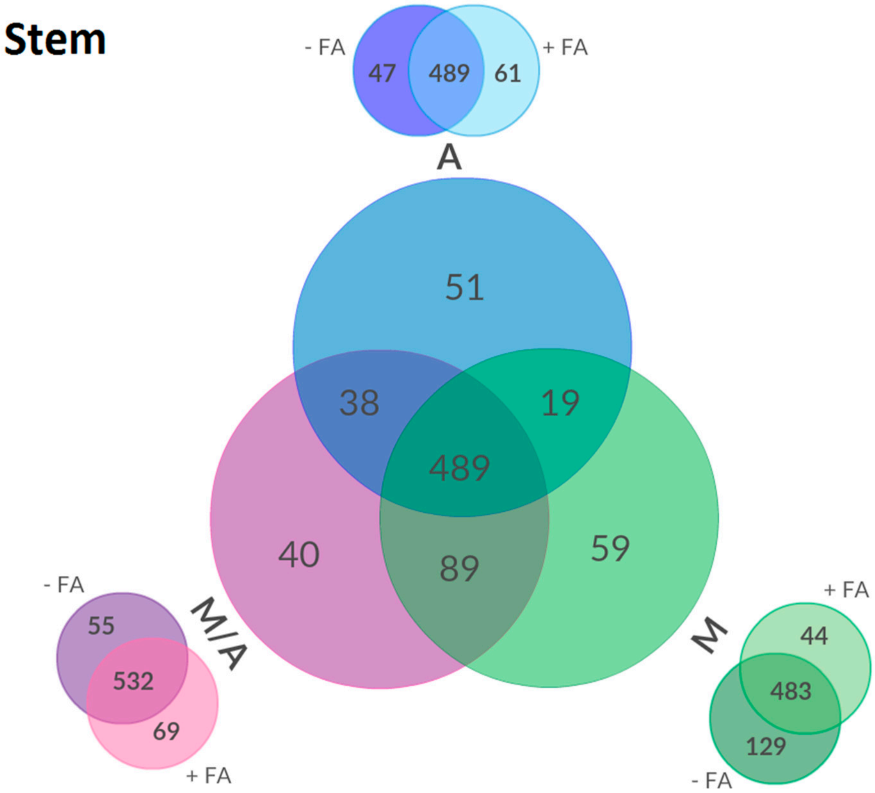

Figure S3. Venn diagram of metabolites detected in wheat stem samples.

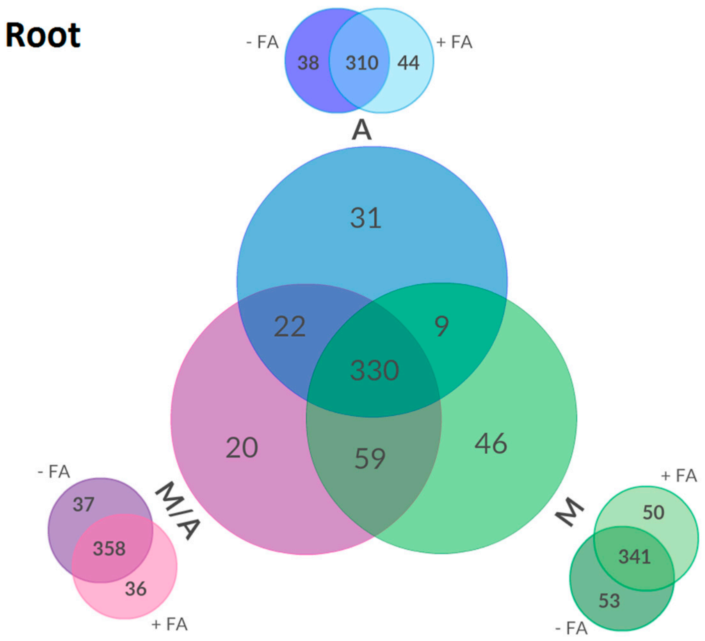

Figure S4. Venn diagram of metabolites detected in wheat root samples.

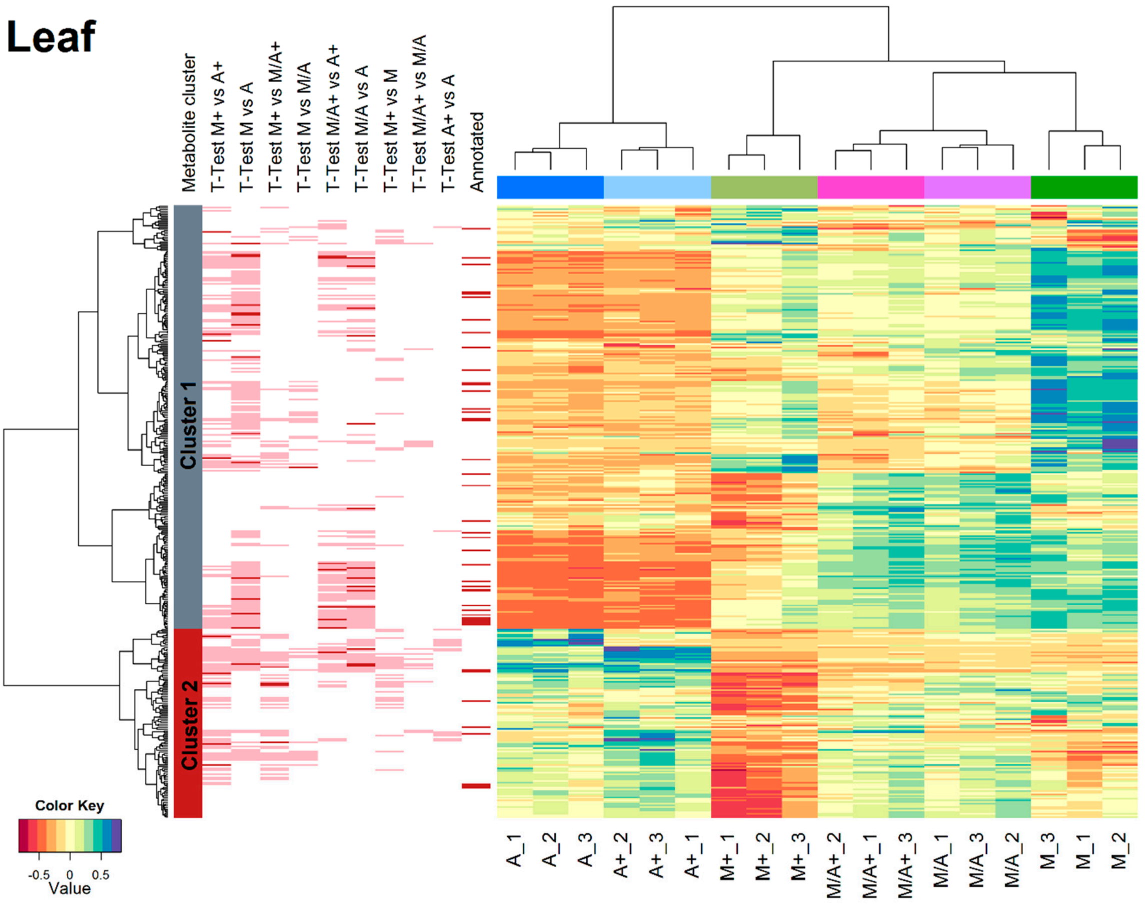

Figure S5. Heatmap of metabolites detected in all tested extraction mixtures in leaf samples.

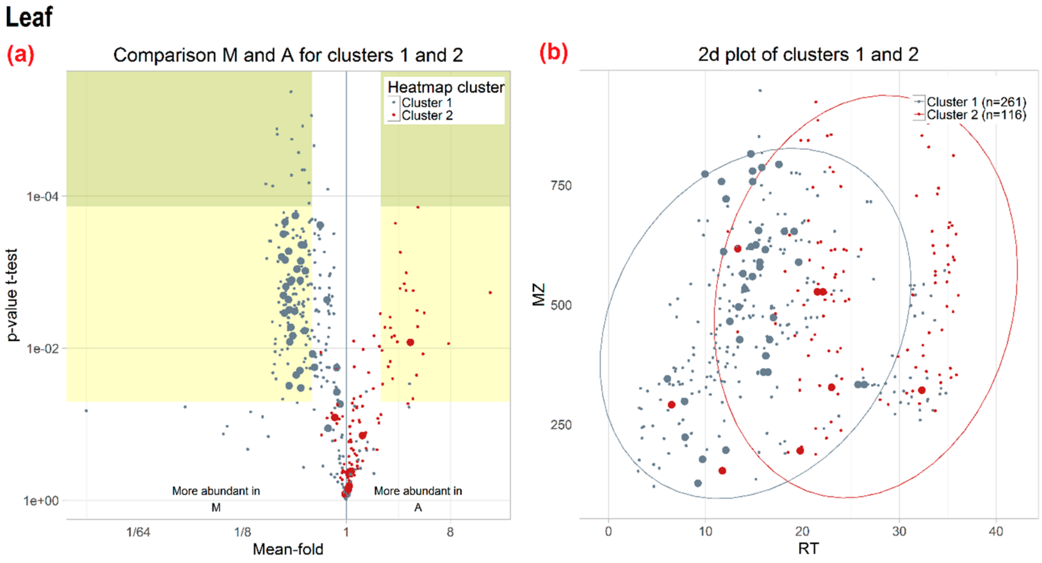

Figure S6. Volcano plot (**a**) and 2D plot of retention times and *m*/*z* values (**b**) of leaf samples.

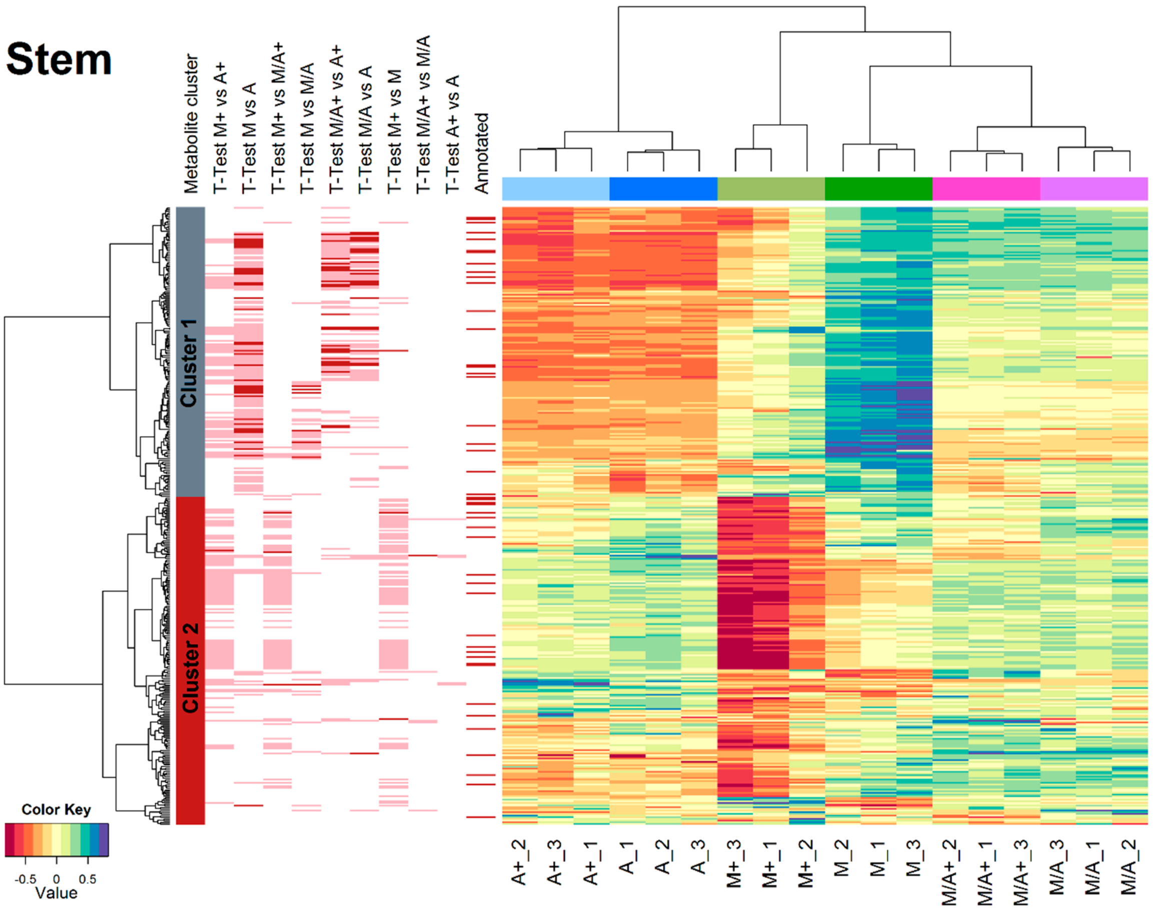

Figure S7. Heatmap of metabolites detected in all tested extraction mixtures in stem samples.

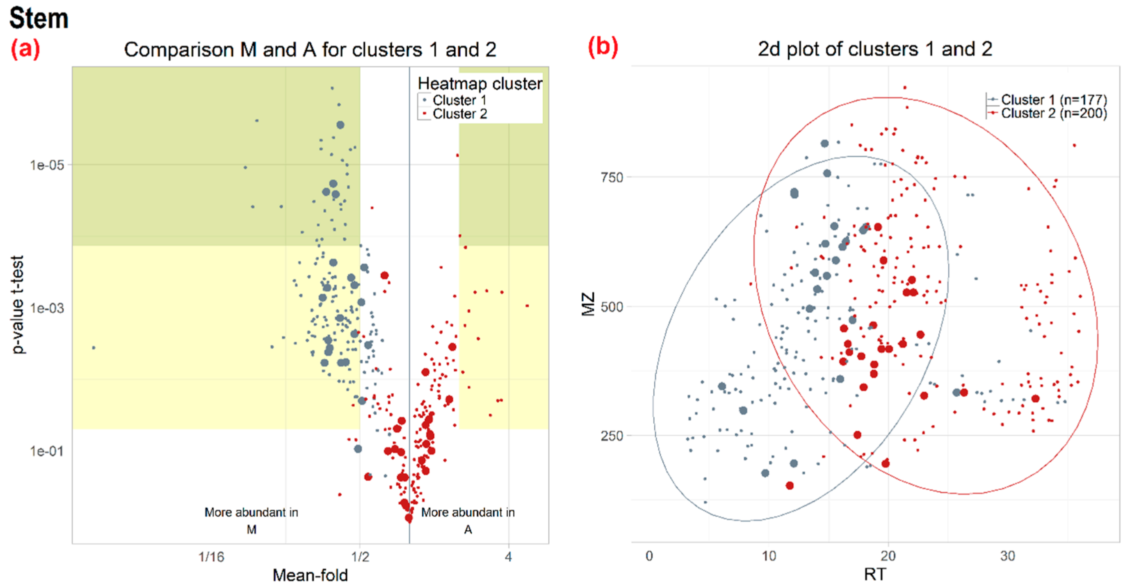

Figure S8. Volcano plot (**a**) and 2D plot of retention times and *m*/*z* values (**b**) of stem samples.

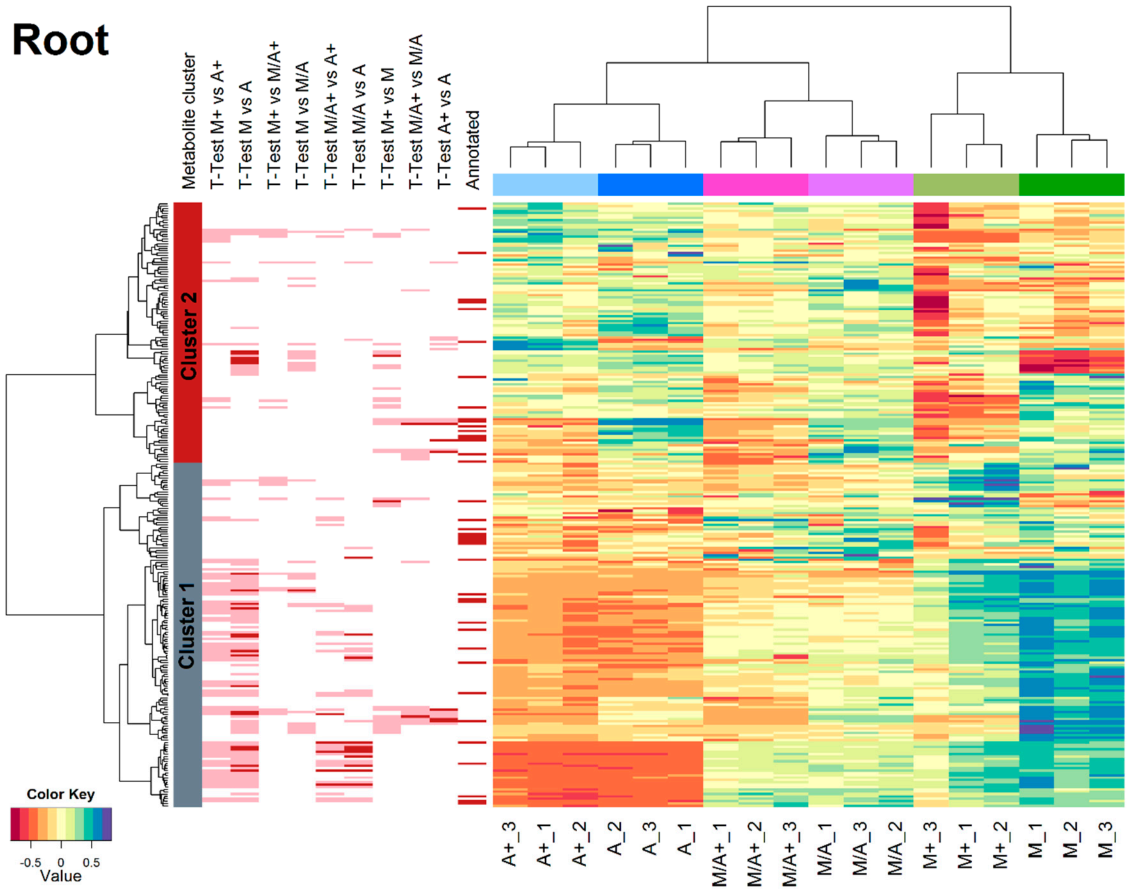

Figure S9. Heatmap of metabolites detected in all tested extraction mixtures in root samples.

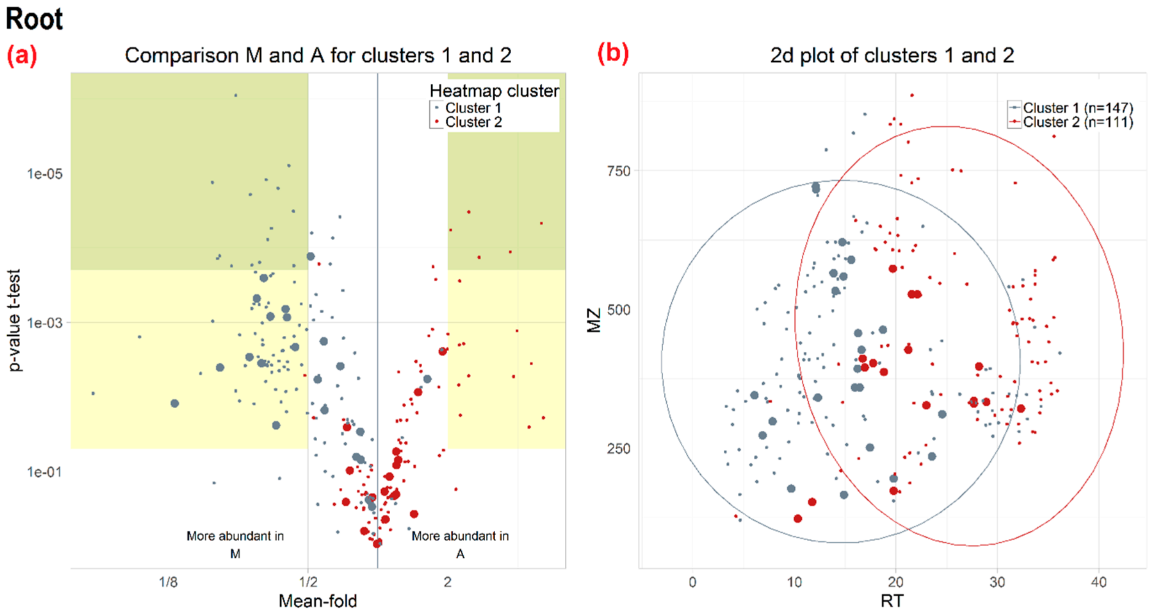

Figure S10. Volcano plot (**a**) and 2D plot of retention times and *m*/*z* values (**b**) of root samples.

Tabe S1. Annotated metabolites.

Metabolite ID	Ion Form	Neutral Mass Known	Retention Time	Accurate Mass	Δ ppm	Number of Carbon Atoms	Name or Substance Class	Molecular Formula	Exact Mass	Number of db Hits	Detected in All Extraction Mixtures			
											In all Ear Samples	In all Stem Samples	In all Leaf Samples	In all Root Samples
1	[M + H]		3.22	215.1394	1.7	10	d-Desthiobiotin	$C_{10}H_{18}N_2O_3$	214.1317	1	x			
2	[M + H]		3.94	166.0863	0.0	9	L-Phenylalanine *	$C_9H_{11}NO_2$	165.0790	5				
3	[M + H]		5.16	220.1184	2.1	9	Pantothenate;	$C_9H_{17}NO_5$	219.1107	1				
4	[M + K]		6.06	345.1310	−4.0	15	Feruloylagmatine	$C_{15}H_{22}N_4O_3$	306.1692	1	x	x	x	x
5	[M + H]		6.22	205.0971	−0.1	11	L-Tryptophan or isomer	$C_{11}H_{12}N_2O_2$	204.0899	1				
6	[M + H]		6.49	251.1390	0.1	13	p-coumaroylhydroxyputrescine or isomer	$C_{13}H_{18}N_2O_3$	250.1317	1				
7	[M + H]		6.49	291.1460	2.9	14	p-coumaroyldehydrohydroxyagmatine	$C_{14}H_{18}N_4O_3$	290.1379	1			x	
8	[M + Na]	x	6.86	273.1209	−0.1	13	p-coumaroylhydroxyputrescine or isomer	$C_{13}H_{18}N_2O_3$	250.1317	1				x
9	[M + Na]	x	6.89	227.0796	2.3	11	L-Tryptophan or isomer	$C_{11}H_{12}N_2O_2$	204.0899	1				
10	[M + Na]	x	7.51	227.0792	0.3	11	L-Tryptophan *	$C_{11}H_{12}N_2O_2$	204.0899	1				
11	[M + H]		7.56	235.1443	0.7	13	p-coumaroylputrescine	$C_{13}H_{18}N_2O_2$	234.1368	1				
12	[M + H]		7.84	298.0975	2.2	11	5′-Deoxy-5′-(methylthio)adenosine *	$C_{11}H_{15}N_5O_3S$	297.0896	1	x	x	x	x
13	$[M + NH_4]$		7.88	223.1080	1.2	11	5-Methoxy-3-indoleacetic acid or DL-Indole-3-lactic acid	$C_{11}H_{11}NO_3$	205.0739	2	x		x	
14	[M + H]		8.32	293.1612	1.4	14	p-coumaroylhydroxyagmatine	$C_{14}H_{20}N_4O_3$	292.1535	1				
15	[M + H]		9.19	127.0391	1.0	6	triacetic acid lactone or Phloroglucinol	$C_6H_6O_3$	126.0317	2			x	
16	[M + H]		9.31	773.2135	0.0	33	Flavonoid-trihexoside, e.g., Lucenin-2,3′-*O*-glucoside	$C_{33}H_{40}O_{21}$	772.2062	55				
17	[M + Na]	x	9.69	377.0848	1.3	16	Chlorogenic acid *	$C_{16}H_{18}O_9$	354.0951	1	x	x	x	x
18	[M + H]		9.94	773.2153	2.4	33	Flavonoid-trihexoside, e.g., Lucenin-2,3′-*O*-glucoside	$C_{33}H_{40}O_{21}$	772.2062	55			x	
19	[M + H]		10.32	123.0440	−0.1	7	4-hydroxybenzaldehyde *	$C_7H_6O_2$	122.0368	2				x
20	[M + H]		10.62	197.0811	1.2	10	5-Hydroxyconiferyl alcohol	$C_{10}H_{12}O_4$	196.0736	1				
21	[M + H]		10.71	265.1548	0.6	14	Feruloylputrescine	$C_{14}H_{20}N_2O_3$	264.1474	1	x			
22	[M + H]		11.16	757.2185	−0.1	33	Flavonoid-trihexoside, e.g., Camelliaside A	$C_{33}H_{40}O_{20}$	756.2113	79				
23	[M + H]		11.39	611.1607	0.0	27	Flavonoid-dihexoside, e.g., Annulatin 7-rhamnoside-3′-xyloside	$C_{27}H_{30}O_{16}$	610.1534	99				
24	[M + H]		11.47	581.1500	−0.1	26	Flavonoid-dihexoside, e.g., Lucenin-1	$C_{26}H_{28}O_{15}$	580.1428	58				
25	[M + Na]		11.49	633.1429	0.5	27	Flavonoid-dihexoside, e.g., Annulatin 7-rhamnoside-3′-xyloside	$C_{27}H_{30}O_{16}$	610.1534	83				
26	[M + Na]	x	11.64	779.2016	1.4	33	Flavonoid-trihexoside, e.g., Camelliaside A	$C_{33}H_{40}O_{20}$	756.2113	67			x	
27	[M + H]		11.73	153.0547	0.4	8	Phenolic acid, e.g., Vanillin	$C_8H_8O_3$	152.0473	5	x	x	x	x
28	[M + Na]	x	11.86	633.1431	0.7	27	Flavonoid-dihexoside, e.g., Annulatin 7-rhamnoside-3′-xyloside	$C_{27}H_{30}O_{16}$	610.1534	83			x	

Table S1. *Cont.*

Metabolite ID	Ion Form	Neutral Mass Known	Retention Time	Accurate Mass	Δ ppm	Number of Carbon Atoms	Name or Substance Class	Molecular Formula	Exact Mass	Number of db Hits	Detected in All Extraction Mixtures			
											In all Ear Samples	In all Stem Samples	In all Leaf Samples	In all Root Samples
29	[M + Na]		11.90	559.1792	1.0	26	Tetrahydroxyprenylflavanone-hexoside, e.g., Phellavin	$C_{26}H_{32}O_{12}$	536.1894	4	x			
30	[M + Na]	x	12.04	603.1333	2.1	26	Flavonoid-dihexoside, e.g., Lucenin-1	$C_{26}H_{28}O_{15}$	580.1428	55				
31	[M + H]		12.08	196.0607	1.2	9	2-Carboxy-2,3-dihydro-5,6-dihydroxyindole or Dopaquinone	$C_9H_9NO_4$	195.0532	2	x	x	x	
32	[M + Na]		12.11	721.2328	1.8	32	Dihydrophelloside	$C_{32}H_{42}O_{17}$	698.2422	1	x	x	x	x
33	[M + H]		12.27	192.0658	1.6	10	5-hydroxyindole-3-acetic-acid	$C_{10}H_9NO_3$	191.0582	1	x			
34	[M + H]		12.32	341.1386	0.7	20	Chalkone, e.g., Bakuchalcone	$C_{20}H_{20}O_5$	340.1311	47				x
35	[M + H]		12.43	581.1502	0.1	26	Flavonoid-dihexoside, e.g., Lucenin-1	$C_{26}H_{28}O_{15}$	580.1428	58				
36	[M + H]		12.51	465.1033	1.2	21	Flavonoid-hexoside, e.g., Quercetin-3-*O*-glucopyranosid	$C_{21}H_{20}O_{12}$	464.0955	70			x	
37	[M + Na]		12.61	587.1369	−0.4	26	Flavonoid-dihexoside, e.g., Artabotryside	$C_{26}H_{28}O_{14}$	564.1479	85				
38	[M + H]		12.89	449.1080	0.4	21	Flavonoid-hexoside, e.g., luteolin 7-*O*-glucoside	$C_{21}H_{20}O_{11}$	448.1006	97				
39	[M + Na]	x	13.10	587.1379	1.3	26	Flavonoid-dihexoside, e.g., Artabotryside	$C_{26}H_{28}O_{14}$	564.1479	85				
40	[M + Na]		13.31	617.1486	1.4	27	Flavonoid-dihexoside, e.g., Datiscin	$C_{27}H_{30}O_{15}$	594.1585	114			x	
41	[M + Na]		13.39	559.1792	1.0	26	Tetrahydroxyprenylflavanone-hexoside, e.g., Phellavin	$C_{26}H_{32}O_{12}$	536.1894	4	x	x	x	
42	[M + H]		13.41	357.1338	1.6	20	Flavonoid, e.g., monotesone A	$C_{20}H_{20}O_6$	356.1260	39	x			
43	[M + H]		13.51	427.1032	2.0	22	Epiafzelechin 3-*O*-gallate or Epigallocatechin 3-*O*-(4-hydroxybenzoate)	$C_{22}H_{18}O_9$	426.0951	2			x	
44	[M + Na]	x	13.72	617.1487	1.6	27	Flavonoid-dihexoside, e.g., Datiscin	$C_{27}H_{30}O_{15}$	594.1585	114				
45	[M + Na]	x	13.86	587.1377	1.0	26	Schaftoside *	$C_{26}H_{28}O_{14}$	564.1479	85	x	x	x	x
46	[M + Na]		14.03	533.1636	1.1	24	Plumerubroside	$C_{24}H_{30}O_{12}$	510.1737	1	x	x	x	x
47	[M + H]		14.66	815.2249	1.0	35	Flavonoid-trihexoside, e.g., Faratroside	$C_{35}H_{42}O_{22}$	814.2168	4		x	x	
48	[M + H]		14.71	787.2086	0.8	37	Flavonoid-dihexoside-hydroxycinnamicacid ester, e.g., Petunoside	$C_{37}H_{38}O_{19}$	786.2007	9	x	x	x	x
49	[M + 2Na-H]	x	14.83	581.1614	1.3	26	Tetrahydroxyprenylflavanone-hexoside, e.g., Phellavin	$C_{26}H_{32}O_{12}$	536.1894	4	x	x	x	x
50	[M + Na]		14.85	779.1800	0.8	36	Flavonoid-dihexoside-hydroxycinnamicacid ester, e.g., Isovitexin 7-*O*-(6‴-caffeoyl)-beta-D-glucopyranoside	$C_{36}H_{36}O_{18}$	756.1902	11			x	
51	[M + H]		14.86	166.0499	0.4	8	N-formylanthranilic acid *	$C_8H_7NO_3$	165.0426	1				x

Table S1. *Cont.*

Metabolite ID	Ion Form	Neutral Mass Known	Retention Time	Accurate Mass	Δ ppm	Number of Carbon Atoms	Name or Substance Class	Molecular Formula	Exact Mass	Number of db Hits	Detected in All Extraction Mixtures			
											In all Ear Samples	In all Stem Samples	In all Leaf Samples	In all Root Samples
52	[M + H]		14.86	757.1983	1.2	36	Flavonoid-dihexoside-hydroxycinnamica cid ester, e.g., Isovitexin 7-*O*-(6‴-caffeoyl) -beta-D-glucopyranoside	$C_{36}H_{36}O_{18}$	756.1902	21		x	x	
53	[M + H]		15.17	655.1871	0.3	29	Flavonoid-dihexoside, e.g., Dillenetin 3,7-diglucoside	$C_{29}H_{34}O_{17}$	654.1796	18				
54	[M + H]		15.19	625.1767	0.7	28	Flavonoid-dihexoside, e.g., 6-*C*-Arabinopyranosyl-8-*C*-glucopyranosyltricin	$C_{28}H_{32}O_{16}$	624.1690	81	x		x	
55	[M + Na]	x	15.46	677.1692	0.5	29	Dimethoxy-tetrahydroxyflavon-dihexoside, e.g., Limocitrin 3-rutinoside	$C_{29}H_{34}O_{17}$	654.1796	13	x	x	x	
56	[M + Na]		15.58	617.1490	2.1	27	Flavonoid-dihexoside, e.g., Datiscin	$C_{27}H_{30}O_{15}$	594.1585	114				
57	[M + Na]	x	15.59	601.1532	0.8	27	Flavonoid-dihexoside, e.g., Chrysin 7-gentiobioside	$C_{27}H_{30}O_{14}$	578.1636	86			x	
58	[M + H]		15.59	589.1920	0.7	29	rotenoid flavonoids, e.g., Dalbin	$C_{29}H_{32}O_{13}$	588.1843	4	x	x	x	x
59	[M + Na]	x	15.80	809.1907	0.9	37	Flavonoid-dihexoside-hydroxycinnamica cid ester, e.g., Isoorientin 4′-*O*-glucoside 2″-*O*-(*E*)-ferulate	$C_{37}H_{38}O_{19}$	786.2007	6			x	
60	[M + Na]	x	15.96	427.1367	0.8	21	Dihydroxyflavan-hexoside, e.g., Koaburanin	$C_{21}H_{24}O_{8}$	404.1471	3	x	x	x	x
61	[M + Na]		16.14	823.2278	1.3	35	Flavonoid-trihexoside, e.g., Tricin 7-rutinoside-4′-glucoside	$C_{35}H_{44}O_{21}$	800.2375	2	x	x	x	
62	[M + Na]	x	16.21	823.2062	0.8	38	Flavonoid-dihexoside-hydroxycinnamicacid ester, e.g., Isorhamnetin 3-(3‴-feruloylrhamnosyl)-(1->6)-galactoside	$C_{38}H_{40}O_{19}$	800.2164	2	x	x	x	x
63	[M + Na]	x	16.26	457.1473	0.8	22	Chalcone, e.g., beta-Hydroxy-2,3,4,5,2′,4',5′-heptamethoxychalcone	$C_{22}H_{26}O_{9}$	434.1577	11	x	x		x
64	[M + Na]		16.42	625.2257	0.3	31	5,4′-Dihydroxy-6-*C*-prenylflavanone 4′-xylosyl-(1->2)-rhamnoside	$C_{31}H_{38}O_{12}$	602.2363	2		x		
65	[M + Na]	x	16.43	793.1957	0.9	37	Flavonoid-dihexoside-hydroxycinnamicacid ester, e.g., 6‴-Feruloylsaponarin	$C_{37}H_{38}O_{18}$	770.2058	6	x		x	x
66	[M + Na]	x	16.60	629.1845	0.7	29	Flavonoid-dihexoside, e.g., Embinin	$C_{29}H_{34}O_{14}$	606.1949	7	x	x	x	x
67	[M + Na]		16.72	411.1418	0.8	21	2,3,4,2′,4′,6′-Hexamethoxychalcone or Sphenostylin C	$C_{21}H_{24}O_{7}$	388.1522	2	x	x		x

Table S1. *Cont.*

Metabolite ID	Ion Form	Neutral Mass Known	Retention Time	Accurate Mass	Δ ppm	Number of Carbon Atoms	Name or Substance Class	Molecular Formula	Exact Mass	Number of db Hits	Detected in All Extraction Mixtures			
											In all Ear Samples	In all Stem Samples	In all Leaf Samples	In all Root Samples
68	[M + Na]	x	16.92	395.1104	0.8	20	Flavonoid, e.g., Asplenetin	$C_{20}H_{20}O_7$	372.1209	53	x			x
69	[M + H]		16.99	829.2404	0.8	36	Isorhamnetin 3-[2″-(4‴-acetylrhamnosyl)-gentiobioside]	$C_{36}H_{44}O_{22}$	828.2324	1	x	x	x	
70	[M + Na]		17.34	365.0996	0.1	19	Flavonoid, e.g., 5,6,7,8-Tetramethoxyflavone	$C_{19}H_{18}O_6$	342.1103	40				
71	[M + Na]	x	17.41	425.1209	0.5	21	Hexamethoxyflavone, e.g., Bannamurpanisin	$C_{21}H_{22}O_8$	402.1315	30	x	x		x
72	[M + Na]		17.56	793.1956	0.7	37	Flavonoid-dihexoside-hydroxycinnamica cid ester, e.g., 6‴-Feruloylsaponarin	$C_{37}H_{38}O_{18}$	770.2058	6			x	
73	[M + H]		17.73	551.1915	0.6	30	Flavanol, e.g., Rel-5-hydroxy-7,4′-dimethoxy-2″ S-(2,4,5-trimethoxy-E-styryl)-tetrahydrofuro [4″R,5″R:2,3]flavanonol	$C_{30}H_{30}O_{10}$	550.1839	4				
74	[M + H]		17.74	403.1389	0.4	21	Hexamethoxyflavone, e.g., Bannamurpanisin	$C_{21}H_{22}O_8$	402.1315	30	x	x		x
75	[M + Na]		17.88	647.1590	1.2	28	Methoxy-trihydroxyflavanol-dihexoside or dimethoxy, trihydroxyflavanol-dihexoside, e.g., 6-*C*-Arabinopyranosyl-8-*C*-glucopyranosyltricin	$C_{28}H_{32}O_{16}$	624.1690	75	x	x		
76	[M + H]		17.92	343.1180	1.0	19	Flavonoid, e.g., 5,6,7,8-Tetramethoxyflavone	$C_{19}H_{18}O_6$	342.1103	40	x	x		
77	[M + H]		18.13	653.1718	0.9	29	Flavonoid-dihexoside, e.g., Isorhamnetin 3-(2‴-acetyl-alpha-arabinopyranosyl)—(1->6)-galactoside	$C_{29}H_{32}O_{17}$	652.1640	15		x	x	
78	[M + Na]		18.72	463.1844	0.9	24	Di-feruloylputrescine	$C_{24}H_{28}N_2O_6$	440.1947	1		x		x
79	[M + H]		18.78	369.1335	0.7	21	Flavonoid, e.g., Gancaonin N	$C_{21}H_{20}O_6$	368.1260	74	x	x		
80	[M + H]		18.81	387.1442	1.1	21	Flavonoid, e.g., Kushenol W	$C_{21}H_{22}O_7$	386.1366	15	x	x		x
81	[M]	x	19.12	737.1601	−1.5	30	Flavonoid-dihexoside, e.g., Cladrastin 7-*O*-laminaribioside	$C_{30}H_{36}O_{16}$	652.2003	4	x	x	x	
82	[M + H]		19.42	417.1544	0.1	22	Flavonoid, e.g., Aliarin	$C_{22}H_{24}O_8$	416.1471	5	x	x		
83	[M + Na]		19.60	589.1531	0.6	26	Flavonoid-dihexoside, e.g., Butein 4′-arabinosyl-(1->4)-galactoside	$C_{26}H_{30}O_{14}$	566.1636	8	x	x	x	
84	[M + Na]	x	19.69	573.1736	0.9	30	Flavanol, e.g., Rel-5-hydroxy-7,4′-dimethoxy-2″S-(2,4,5-trimethoxy-E-styryl)-tetrahydrofuro [4″R,5″R:2,3]flavanonol	$C_{30}H_{30}O_{10}$	550.1839	4	x			x

Table S1. *Cont.*

Metabolite ID	Ion Form	Neutral Mass Known	Retention Time	Accurate Mass	Δ ppm	Number of Carbon Atoms	Name or Substance Class	Molecular Formula	Exact Mass	Number of db Hits	Detected in All Extraction Mixtures			
											In all Ear Samples	In all Stem Samples	In all Leaf Samples	In all Root Samples
85	[M + Na]		19.76	195.0993	0.6	9	9-Oxononanoic acid	$C_9H_{16}O_3$	172.1099	1	x	x	x	x
86	[M + H]		20.06	417.1548	1.0	22	Flavonoid, e.g., Aliarin	$C_{22}H_{24}O_8$	416.1471	5		x		
87	[M + Na]	x	20.21	573.1370	0.4	29	Formononetin 7-*O*-(2″-p-hydroxybenzoylglucoside)	$C_{29}H_{26}O_{11}$	550.1475	1	x			
88	[M + Na]		20.91	423.1417	0.6	22	Flavanone, e.g.,Kenusanone E	$C_{22}H_{24}O_7$	400.1522	19	x			
89	[M + H]		21.22	427.1391	0.9	23	rotenoid flavonoids, e.g., Dalbinol	$C_{23}H_{22}O_8$	426.1315	15	x	x		x
90	[M + K]		21.53	617.1243	−4.0	27	Flavonoid-dihexoside, e.g., Chrysin 7-gentiobioside	$C_{27}H_{30}O_{14}$	578.1636	86	x	x	x	x
91	[M + Na]	x	21.96	573.1372	0.7	29	Formononetin 7-*O*-(2″-p-hydroxybenzoylglucoside)	$C_{29}H_{26}O_{11}$	550.1475	1	x	x		
92	[M + K]		22.09	617.1242	−4.0	27	Flavonoid-dihexoside, e.g., Chrysin 7-gentiobioside	$C_{27}H_{30}O_{14}$	578.1636	86	x	x	x	x
93	[M + H]		22.68	445.1132	0.7	22	Flavonoid-hexoside, e.g., 3′-*O*-Methylderhamnosylmaysin	$C_{22}H_{20}O_{10}$	444.1057	4	x	x		
94	[M + Na]		22.99	573.1736	0.8	30	Flavanol, e.g., Rel-5-hydroxy-7,4′-dimethoxy-2″*S*-(2,4,5-trimethoxy-E-styryl) -tetrahydrofuro[4″R,5″R:2,3]flavanonol	$C_{30}H_{30}O_{10}$	550.1839	4		x	x	x
95	[M + Na]		23.53	235.1310	2.1	12	Traumatin;	$C_{12}H_{20}O_3$	212.1412	1	x			x
96	[M + H]		23.56	445.1131	0.4	22	Flavonoid-hexoside, e.g., 3′-*O*-Methylderhamnosylmaysin	$C_{22}H_{20}O_{10}$	444.1057	4	x			
97	[M + Na]	x	24.18	589.1320	0.6	29	Isoporiolide or poriolide	$C_{29}H_{26}O_{12}$	566.1424	2				
98	[M + H]		24.40	285.1124	1.0	17	Chalcone, e.g., Aurentiacin A	$C_{17}H_{16}O_4$	284.1049	28	x			
99	[M + H]		24.55	311.2222	1.5	18	9-Hydroperoxy-10,12,15-octadecatrienoate or isomer	$C_{18}H_{30}O_4$	310.2144	1	x			x
100	[M + 2Na-H]	x	25.72	355.1857	0.2	18	9-Hydroperoxy-10,12,15-octadecatrienoate or isomer	$C_{18}H_{30}O_4$	310.2144	1	x	x	x	
101	[M + CH_3FeN]	x	26.34	395.1744	−2.4	18	9-Hydroperoxy-10,12,15-octadecatrienoate or isomer	$C_{18}H_{30}O_4$	310.2144	1	x	x	x	
102	[M + Na]	x	26.63	335.2195	0.6	18	octadecanoic acid derivatives, e.g., (9*Z*,11*E*)-(13*S*)-13-Hydroperoxyoctadeca-9,11-dienoic acid	$C_{18}H_{32}O_4$	312.2301	4				
103	[M+Na]		27.65	335.2199	1.9	18	octadecanoic acid derivatives, e.g., (9*Z*,11*E*)-(13*S*)-13-Hydroperoxyoctadeca-9,11-dienoic acid	$C_{18}H_{32}O_4$	312.2301	4	x			x

Table S1. *Cont.*

Metabolite ID	Ion Form	Neutral Mass Known	Retention Time	Accurate Mass	Δ ppm	Number of Carbon Atoms	Name or Substance Class	Molecular Formula	Exact Mass	Number of db Hits	Detected in All Extraction Mixtures			
											In all Ear Samples	In all Stem Samples	In all Leaf Samples	In all Root Samples
104	[M + NH_4]		27.67	330.2644	1.7	18	octadecanoic acid derivatives, e.g., (9*Z*,11*E*)-(13*S*)-13-Hydroperoxyoctadeca-9,11-dienoic acid	$C_{18}H_{32}O_4$	312.2301	4	x			x
105	[M + H]		28.09	297.2424	0.0	18	octadecanoic acid derivatives, e.g., 9(*S*)-HODE	$C_{18}H_{32}O_3$	296.2351	2				
106	[M + CH_3FeN]	x	28.17	397.1900	−2.4	18	octadecanoic acid derivatives, e.g., (9*Z*,11*E*)-(13*S*)-13-Hydroperoxyoctadeca-9,11-dienoic acid	$C_{18}H_{32}O_4$	312.2301	4	x			x
107	[M + Na]		28.90	333.2043	2.1	18	9-Hydroperoxy-10,12,15-octadecatrienoate or isomer	$C_{18}H_{30}O_4$	310.2144	1				x
108	[M + Na]		32.31	321.2403	0.9	18	*cis*-9,10-Epoxystearic acid	$C_{18}H_{34}O_3$	298.2508	1	x	x	x	x
109	[M + NH_4]		34.56	711.2394	−0.2	35	Licorice glycoside E	$C_{35}H_{35}NO_{14}$	693.2058	1				

*: confirmed with authentic reference standard.
